# TB-YOLOv8n: an improved YOLOv8n-based model for tip-burn detection in plant factory-grown pakchoi under LED lighting

**DOI:** 10.3389/fpls.2026.1933084

**Published:** 2026-09-23

**Authors:** Peng Wang, Wenwen Hu, Xiang Ma, Shipu Xu, Zhiwen Zhou, Yunsheng Wang

**Affiliations:** 1 College of Information Technology, Shanghai Ocean University, Shanghai, China; 2 Institute of Agricultural Information Science Technology, Shanghai Academy of Agricultural Sciences, Shanghai, China; 3 Key Laboratory of Intelligent Agricultural Technology (Yangtze River Delta), Ministry of Agriculture and Rural Affairs, Shanghai, China

**Keywords:** computer vision, lightweight model, object detection, pakchoi, plant factory, red-blue LED lighting, tip-burn, YOLOv8

## Abstract

**Introduction:**

Plant factory-grown pakchoi is prone to tip-burn under high-density cultivation, limited airflow, and rapid growth conditions, which reduces leaf integrity and commercial quality. Image-based detection of small or mild visible tip-burn symptoms remains challenging because the affected regions are usually small, irregularly shaped, low in color contrast, and easily disturbed by red-blue LED lighting, leaf overlap, curled leaf margins, and heart-leaf occlusion.

**Methods:**

To address these challenges, this study constructed a pakchoi tip-burn image dataset under plant factory conditions and proposed TB-YOLOv8n, a lightweight detection model based on YOLOv8n. AFGCAttention was introduced at the end of the backbone to enhance informative channel responses related to subtle tip-burn regions and suppress redundant background information. GLSA was embedded between the backbone and neck to integrate local texture details with global spatial context under leaf overlap and low-contrast symptom conditions. In addition, a P2-enhanced BiFPN neck was constructed to strengthen cross-scale feature fusion and preserve fine spatial information for small visible tip-burn regions.

**Results:**

Experimental results showed that TB-YOLOv8n achieved a precision of 89.4%, a recall of 87.6%, an mAP@0.5 of 93.2% ± 0.3%, and an mAP@0.5–0.95 of 59.0% ± 0.3%, which were 4.2, 3.9, 3.4, and 10.0 percentage points higher than those of the original YOLOv8n, respectively. The model contained only 2.2 M parameters, required 7.6 G FLOPs, had a model size of 4.8 MB, and achieved a model-forward inference speed of 138 FPS on the RTX 4090 and 33.0 FPS on the Jetson AGX Orin. Ablation experiments, heatmap visualization, and comparisons with mainstream detection models further confirmed the effectiveness of the proposed improvements.

**Discussion:**

The results indicate that TB-YOLOv8n achieves a favorable balance among detection accuracy, lightweight structure, and real-time inference capability. The proposed model provides a basis for visible tip-burn monitoring and subsequent production decision support in plant factories.

## 1 Introduction

Facility agriculture is increasingly being combined with information technology to improve crop production efficiency and management quality. Plant factories represent a typical form of this development, as they use artificial lighting, controlled environments, and vertical cultivation systems to produce vegetables throughout the year in limited space (Guo et al., 2026). Leafy vegetables are important crops in greenhouse and controlled-environment production systems, accounting for about 30-40% of total vegetable production (Liang et al., 2025). Among them, pakchoi (Brassica rapa subsp. chinensis) is widely grown in Asia, especially in China, due to its short growth cycle and good adaptability to dense cultivation (Wu et al., 2025; Zhou et al., 2025). Light is one of the key factors affecting pakchoi growth in plant factories. Red-blue LED lighting can regulate photosynthesis and plant morphology (Mahesh et al., 2025). However, under dense planting and poor airflow, fast-growing young leaves may suffer from an imbalance in calcium supply, which can lead to tip-burn at leaf tips and margins (Ertle and Kubota, 2025). High light intensity and uneven illumination may also change the canopy environment and leaf transpiration, further increasing the risk of tip-burn (Yu et al., 2024; Moosavi-Nezhad and Meng, 2025).

Tip-burn, also known as dry heart, is a common physiological disorder of leafy vegetables in controlled environments, rather than a disease caused by pathogen infection (Uno et al., 2016; Lin et al., 2026). At the onset of visible symptoms, slight chlorosis, shrinkage, or water-soaked areas usually appear at leaf tips or margins. As the disorder develops, the affected areas gradually become brown and necrotic, forming dry and papery lesions that reduce leaf integrity and commercial value (Simko and Ospina-Giraldo, 2026). At present, tip-burn detection still largely relies on manual inspection and visual judgment (Gozzovelli et al., 2021; Kumaratenna and Cho, 2024). However, under multi-layer vertical cultivation, severe leaf overlap, and occlusion, manual inspection is difficult to perform comprehensively, and small or mild visible symptoms are often missed. Therefore, developing an automatic, rapid, and accurate method for tip-burn detection is essential for improving the intelligent management of plant factories (Uno et al., 2016; Hamidon and Ahamed, 2022).

Computer vision technology has been increasingly applied to plant disease recognition and physiological stress monitoring because of its advantages of non-contact operation, low damage, continuous image acquisition, and ease of automated deployment (Paul et al., 2025). Story et al. (2010) investigated early detection of calcium deficiency in greenhouse lettuce using a machine vision system and extracted handcrafted features, including projected canopy area, color, and texture. Their method provided warning one day before symptoms became visible to the human eye, demonstrating the potential of image features for early monitoring of calcium deficiency stress in leafy vegetables. Lu et al. (2023) applied traditional machine learning algorithms, including K-nearest neighbor, support vector machine, and random forest, to detect potassium, calcium, and magnesium deficiency symptoms in lettuce under LED lighting. They further compared texture-feature-based methods with lightweight deep learning models, showing that deep learning models generally outperformed traditional machine learning approaches. However, traditional image analysis methods usually rely on manually designed color, texture, and morphological features, and their performance is highly affected by feature selection and imaging conditions (Javidan et al., 2024). In plant factories, colored light sources, local shadows, and leaf overlap can make these features unstable, which increases the difficulty of symptom recognition.

Deep learning has become an important method for plant stress and disease diagnosis in recent years (Mohanty et al., 2016). Convolutional neural networks can learn image features directly, which reduces the need for manual feature design and improves adaptability to complex scenes (Boulent et al., 2019). Shaha et al. (2025) used CNN models to classify healthy and tip-burn lettuce leaves grown in indoor hydroponic systems, achieving accuracies of 98% and 95%, respectively, which demonstrated the potential of CNNs for indoor leafy vegetable tip-burn recognition. Li et al. (2025) investigated calcium deficiency and tip-burn-related symptom detection in hydroponic lettuce under colored LED lighting. They used U-Net++ for leaf segmentation and EfficientNetB2 for calcium deficiency classification, achieving segmentation accuracy of 98.56% and classification accuracy of 91.51%, respectively. However, such methods mainly determine whether a plant or image contains calcium deficiency symptoms and have limited ability to provide the spatial location of affected regions. Franchetti and Pirri (2022) proposed a method combining classification and self-supervised segmentation for tip-burn detection in high-density lettuce canopies grown in plant factories. Their method localized and finely segmented small tip-burn regions from canopy images containing approximately 1,000 plants, improving symptom identification in dense backgrounds. Nevertheless, this approach focuses more on fine segmentation and phenotypic analysis, with a relatively complex workflow, which may limit its suitability for real-time deployment.

Compared with classification methods that only output category labels and fine segmentation methods with complex processing pipelines, object detection models can simultaneously provide symptom categories and spatial locations, achieving a better balance among detection accuracy, inference speed, and deployment cost. Yang et al. (2025) proposed AgriLiteNet for tomato pest and disease detection by integrating lightweight CNN–Transformer feature extraction with multi-scale feature fusion, and further validated its deployment on an NVIDIA Jetson Orin NX platform. The YOLO series has been widely used in agricultural disease detection and crop health monitoring because of its end-to-end training, fast detection speed, and convenient deployment (Badgujar et al., 2024). Wang and Liu (2024) proposed a YOLOv8n-vegetable model for greenhouse vegetable disease detection and improved detection performance with a lightweight feature extraction module. Zhu et al. (2026) developed a YOLOv8n-based model for pakchoi pest and disease detection, improving disease-region recognition by enhancing feature extraction and multi-scale feature fusion. To address the challenges of small lesion size, irregular lesion morphology, and strong background interference, Abudukelimu et al. (2025) proposed an improved YOLOv8 model for cotton leaf disease detection, reducing missed and false detections of small lesions by strengthening small-target feature representation and attention modeling. Zheng et al. (2024) proposed YOLOPC based on YOLOv5s for pakchoi pest and disease identification. Their model achieved an mAP of 91.4%, which was 12.9 percentage points higher than that of the original YOLOv5s.

Several studies have also explored tip-burn detection in indoor crop production. Hamidon and Ahamed (2022) compared CenterNet, YOLOv4, and YOLOv5 for lettuce tip-burn detection under white, red, and blue LED lighting. YOLOv5 achieved the best performance, with an mAP of 82.8%, showing that YOLO-based models can detect tip-burn under artificial lighting. Chutichaimaytar et al. (2025) proposed the Coriander Tip-Burn YOLO (CTB-YOLO) model for early detection of tip-burn and powdery mildew in indoor coriander. By using multi-scale feature fusion, CTB-YOLO improved the detection of small symptomatic regions and was deployed on an edge computing device for real-time warning. The reported mAP values were 76.1% for tip-burn and 69.3% for powdery mildew.

Despite these advances, tip-burn detection in plant factory-grown pakchoi presents distinct challenges. Pakchoi differs from lettuce and coriander in leaf shape, canopy structure, and symptom appearance. Its leaves are more likely to curl, wrinkle, and overlap. Visible tip-burn regions, particularly small or mild symptoms, are often irregular and low in color contrast. They can be confused with normal leaf margins, vein textures, shadows, and reflective areas. Under red-blue LED lighting, image color shifts further reduce the visual difference between healthy and affected tissues. These factors make stable detection of small tip-burn regions difficult.

To address these challenges, this study proposes TB-YOLOv8n, a task-oriented lightweight detection model based on YOLOv8n for tip-burn detection in plant factory-grown pakchoi under red-blue LED lighting. The main contributions are as follows:

1. A dedicated image dataset for pakchoi tip-burn detection in plant factories was constructed. The dataset covers red-blue LED lighting, leaf occlusion, curled leaf margins, and diverse visible tip-burn symptoms. The dataset supports the detection of small visible tip-burn regions under controlled-environment conditions.
2. To enhance the weak visual features of subtle visible tip-burn regions, AFGCAttention was introduced into the YOLOv8n backbone. This module strengthens useful channel responses and suppresses redundant background information, helping the model focus on small affected regions near leaf margins and heart leaves.
3. To improve feature representation under leaf overlap and heart-leaf occlusion, GLSA was used to replace the original channel-alignment convolution in the neck network. By combining global spatial information with local detail features, GLSA helps the model distinguish tip-burn regions from normal leaf textures, shadows, and reflective areas.
4. To preserve shallow spatial details such as edges, textures, and positional information for small tip-burn regions, an improved BiFPN-P2 structure was designed for multi-scale feature fusion. This structure introduces high-resolution P2 features into the fusion process, allowing the model to better retain fine spatial information while maintaining a lightweight design.

## 2 Materials and methods

### 2.1 Dataset construction

#### 2.1.1 Plant materials and cultivation conditions

The experiment was conducted in a plant factory cultivation room at the Shanghai Academy of Agricultural Sciences from September 2025 to February 2026. Two types of hydroponic systems were used: a rotary-moving hydroponic device and a static hydroponic device (**Figure 1**). Both systems used rockwool seedling blocks and net cups for transplanting. As shown in **Figure 1a**, the rotary-moving hydroponic device contained 22 evenly spaced cultivation units. Each unit could hold up to nine pakchoi plants, giving a maximum capacity of 198 plants per cultivation cycle. During cyclic movement, the containers tilted when passing through the sloped section. This movement allowed nutrient solution exchange approximately every 3 min, helping maintain solution flow and dissolved oxygen supply around the roots. The static hydroponic device, shown in **Figure 1b**, contained six nutrient solution circulation units. Each unit had three layers, and each layer could hold six plants. A water pump continuously circulated the nutrient solution to supply water, nutrients, and dissolved oxygen to the root zone. This system could cultivate up to 108 pakchoi plants per cycle.

**Figure 1:**
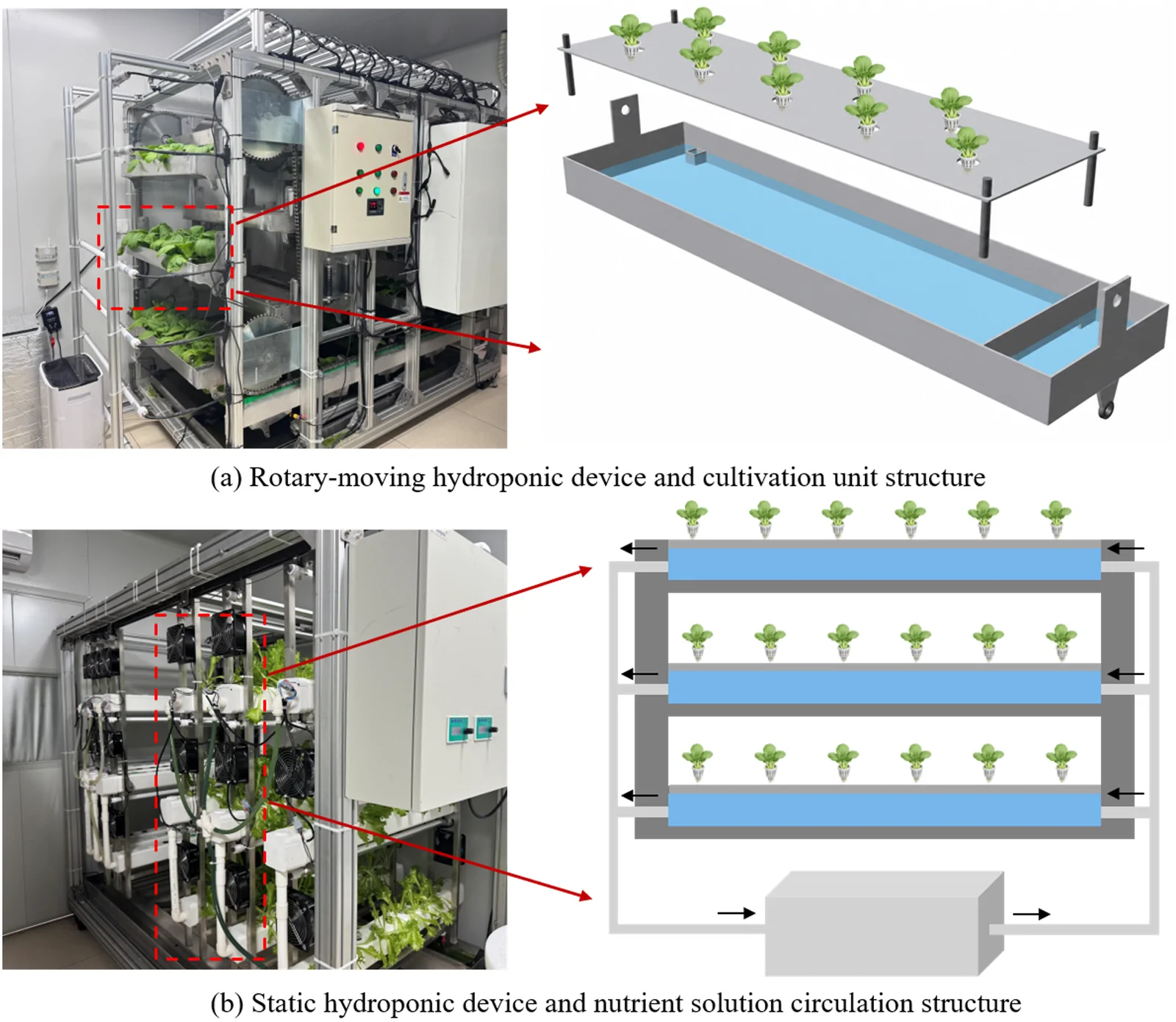
Hydroponic cultivation devices and structural diagrams used for pakchoi cultivation in a plant factory. **(a)** Rotary-moving hydroponic device and cultivation unit structure; **(b)** static hydroponic device and nutrient solution circulation structure.

The pakchoi cultivars used in this study were Yanqing (the Shanghai Academy of Agricultural Sciences) and Jinpin (Fujian Jinpin Agricultural Technology Co., Ltd.) (**Figure 2**). Yanqing has relatively broad and thick leaves, with obvious leaf surface wrinkles and curled leaf margins, whereas Jinpin has relatively flat leaves and a compact heart-leaf structure. Before sowing, the rockwool seedling blocks were fully soaked in water trays. Plump seeds were selected and placed into the center holes of the rockwool blocks, followed by germination in a constant-temperature dark incubator for 48 h. After radicle emergence, the seedlings were transferred to a seedling rack for further cultivation. During the seedling stage, the nutrient solution was maintained at a pH of 6.0 ± 0.5 and an electrical conductivity (EC) of 1.1 ± 0.1 mS/cm. After approximately 14 days, seedlings with uniform growth were selected and transplanted into the hydroponic systems.

**Figure 2:**
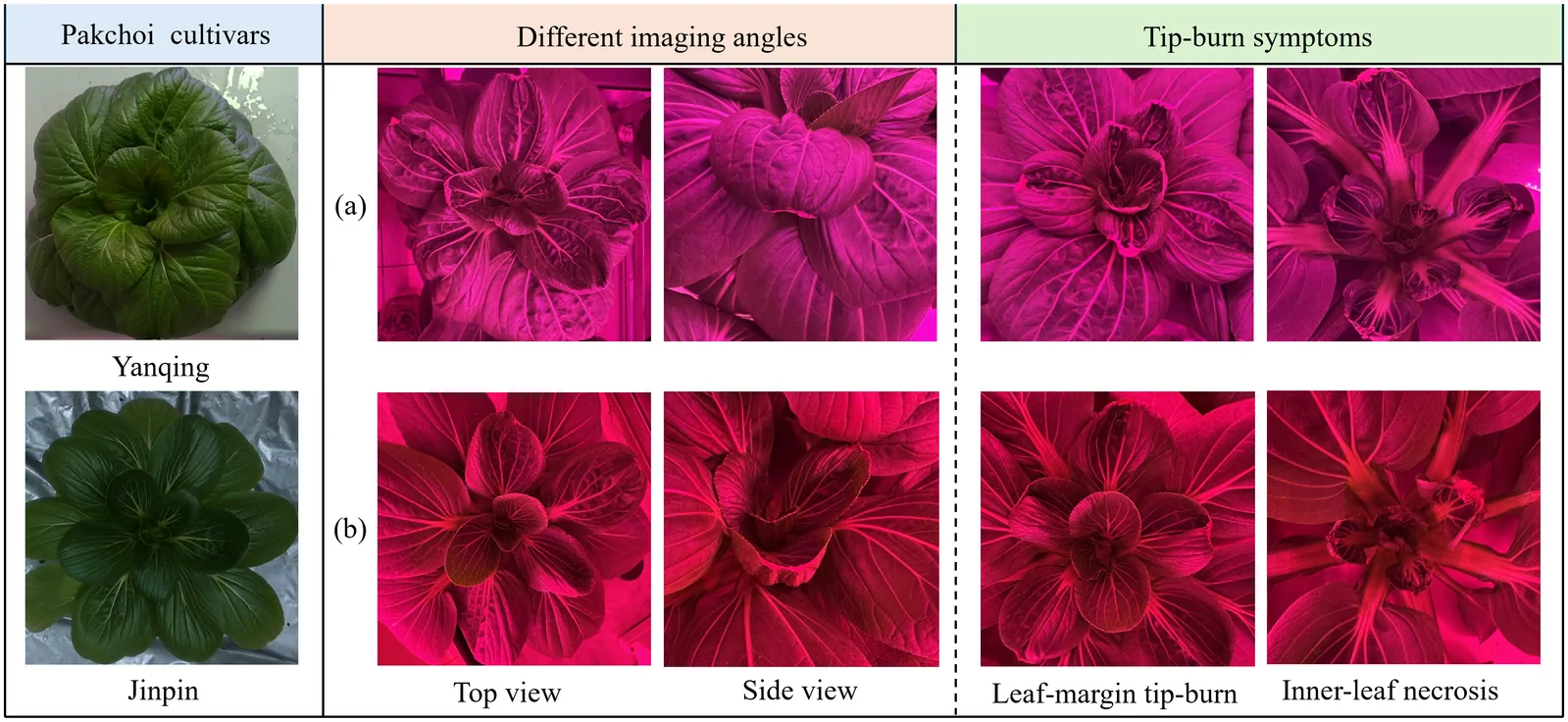
Examples of pakchoi cultivars, imaging angles, and tip-burn symptoms. **(a)** Yanqing under the red–blue light ratio of 8:2; **(b)** Jinpin under the red–blue light ratio of 9:1.

During cultivation, both hydroponic systems were placed under red-blue LED lighting, with a photoperiod of 14 h/day. The plant factory temperature was maintained at 20–23 °C, and the relative humidity was maintained at 60%–70%. During the first 14 days after transplanting, the nutrient solution pH and EC were regularly adjusted to 5.5–6.5 and 1.2–1.5 mS/cm, respectively. In the later cultivation stage, the pH was adjusted to 6.2–7.0, and the EC was increased to 2.0–2.5 mS/cm to obtain more tip-burn samples. The ventilation system was also turned off to increase air humidity, while the occurrence of tip-burn was continuously monitored. Symptom monitoring began 7 days after the induction treatment was initiated. Two cultivation cycles were conducted. In the first cycle, Jinpin was grown in the rotary-moving hydroponic system under a red-blue light ratio of 8:2, whereas Yanqing was grown in the static hydroponic system under a ratio of 9:1. In the second cycle, Yanqing was grown in the rotary-moving system under 8:2, whereas Jinpin was grown in the static system under 9:1. Thus, both cultivars were represented under both red-blue light ratios across the two cultivation cycles. In total, approximately 600 plants were cultivated, of which about 560 developed visible tip-burn symptoms. The full workflow is shown in **Figure 3**.

**Figure 3:**
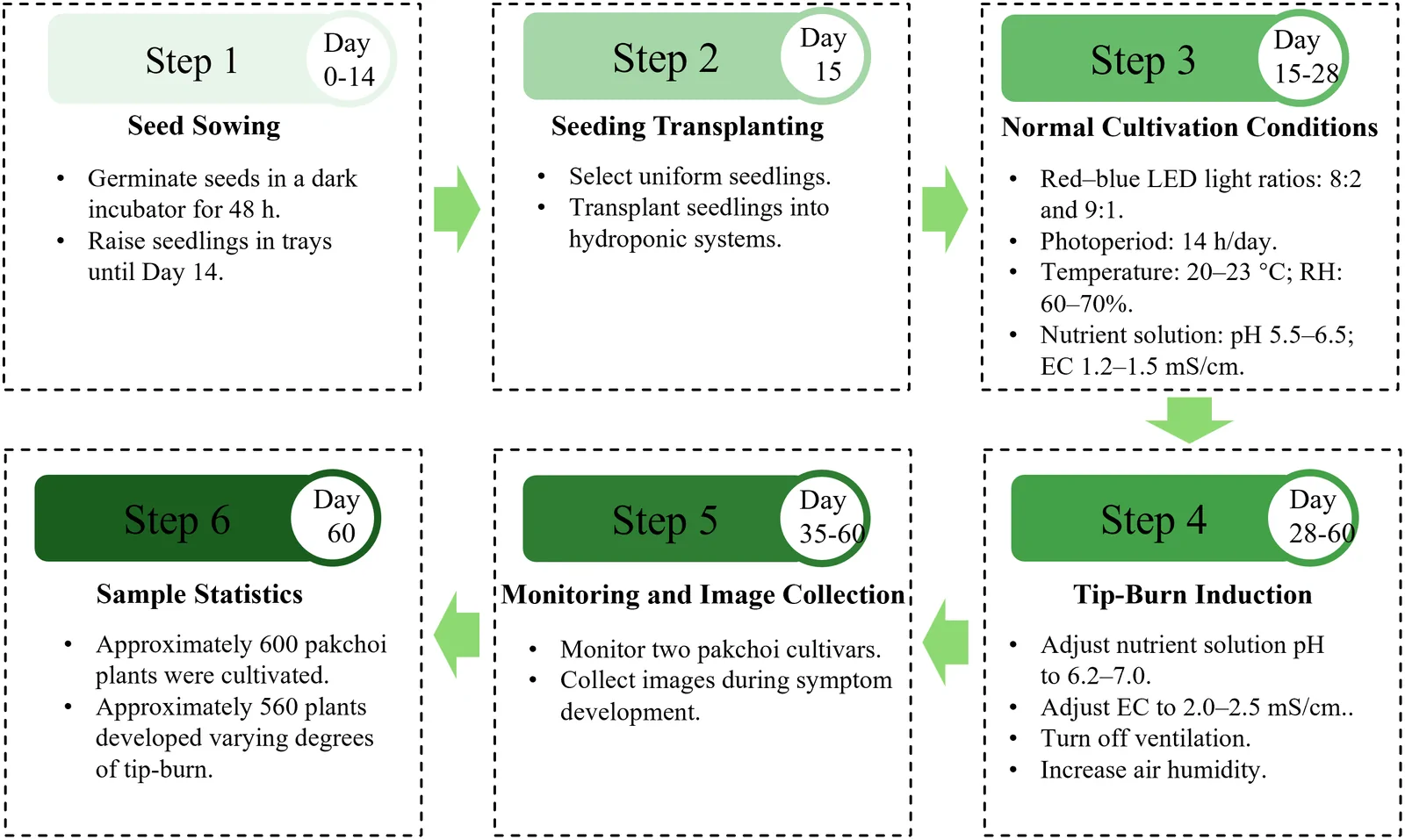
Workflow of pakchoi cultivation, tip-burn induction, and image acquisition in a plant factory.

#### 2.1.2 Data collection and annotation

Image collection started when the first visible tip-burn symptoms appeared and continued until harvest. Images were acquired from different viewing angles using a smartphone camera at an original resolution of 4032 × 3024 pixels. A total of 1,524 images were collected. The first cultivation cycle yielded 571 Jinpin images under the 8:2 red-blue light ratio and 233 Yanqing images under 9:1, whereas the second cycle yielded 501 Yanqing images under 8:2 and 219 Jinpin images under 9:1. **Figure 2** shows representative images of Yanqing and Jinpin pakchoi plants under different red-blue light ratios, including top-view and side-view images with visible tip-burn symptoms. The top-view images provide a more complete view of the canopy structure and show tip-burn symptoms at leaf tips and margins. The side-view images help show leaf overlap, leaf curling, and heart-leaf occlusion, which are difficult to observe from a single angle. Tip-burn symptoms also differed across growth stages. During the leaf expansion stage, symptoms mainly appeared as localized wilting at leaf tips or margins. During the heart-leaf stage, necrosis near the heart leaves or inward tip-burn symptoms could also be observed.

The tip-burn regions were manually annotated using LabelImg. The annotations were jointly completed by two annotators who participated in the cultivation experiments, symptom observation, and image acquisition. Visible chlorotic, shriveled, water-soaked, brown, or necrotic tissues at leaf tips, leaf margins, or near the heart leaves were annotated as tip-burn targets, whereas normal leaf margins, veins, shadows, and light reflections were excluded. A tight bounding box was drawn around each clearly visible symptom region, and ambiguous mild or low-contrast regions were jointly reviewed and resolved by consensus. The annotations were saved as TXT files in YOLO format, and each image corresponded to a TXT file with the same name. An example of the annotation process is shown in **Figure 4**. After annotation, all images were standardized to JPG format and resized to 640 × 640 pixels. The dataset contained 4,560 bounding-box annotations and 33 negative images without visible tip-burn targets; detailed statistics are provided in **Supplementary Table S1**. The cultivation-unit-level dataset partitioning, rather than random image-level splitting, is summarized in **Table 1**. All 804 images from the first cultivation cycle were assigned to the training set. For the second cycle, the 22 cultivation units in the rotary-moving system were divided into 7 training, 9 validation, and 6 test units, corresponding to 168, 215, and 118 images, respectively. The six cultivation units in the static system were divided into 3 training, 2 validation, and 1 test units, corresponding to 94, 89, and 36 images, respectively. All images acquired from the same cultivation unit were retained in the same subset. The final dataset contained 1,066 training, 304 validation, and 154 test images, approximately corresponding to a 7:2:1 ratio.

**Figure 4:**
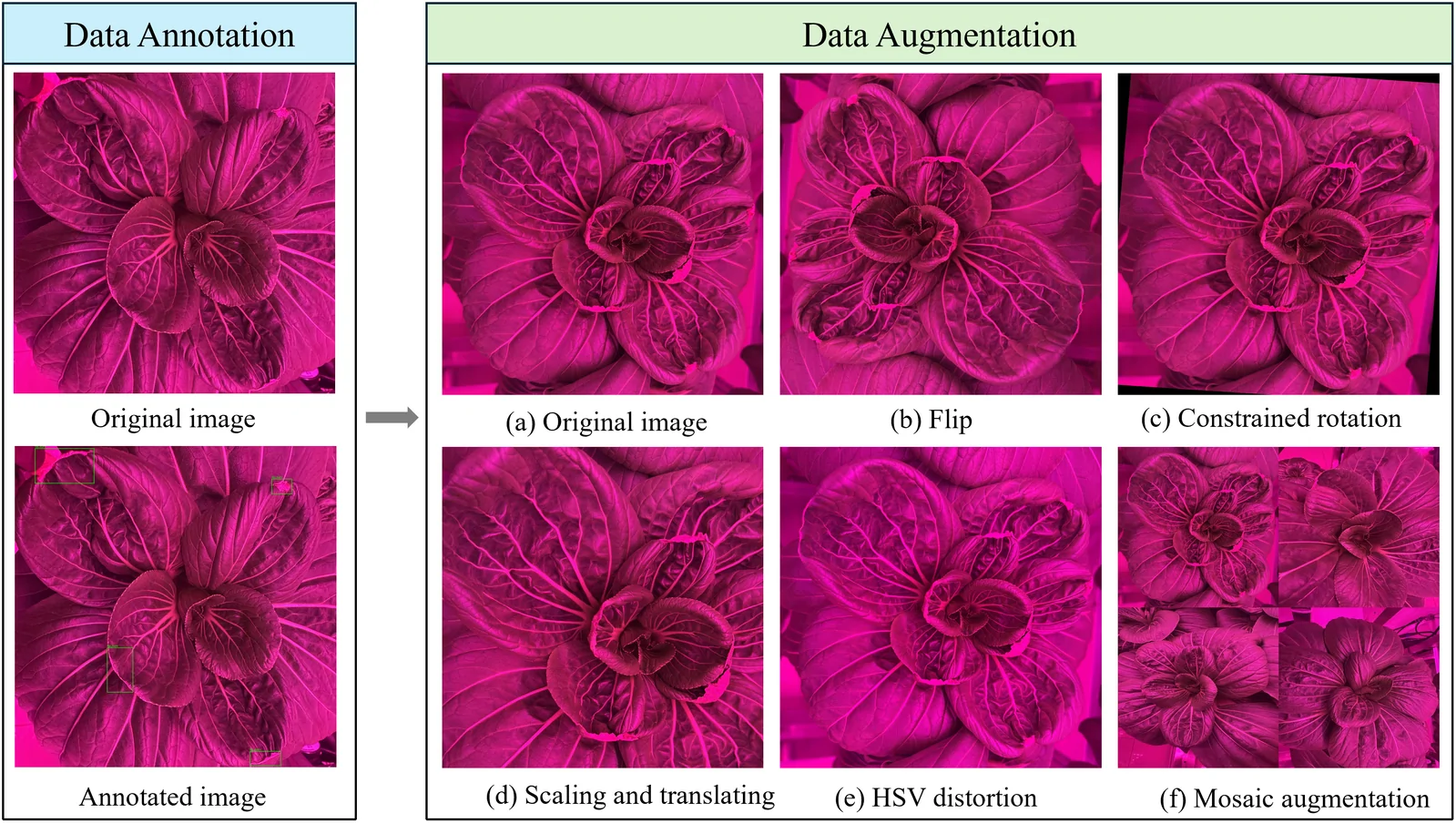
Examples of data annotation and augmentation. The left panel shows the original and annotated images; the right panel shows **(a)** original image, **(b)** flip, **(c)** constrained rotation, **(d)** scaling and translating, **(e)** HSV distortion, and **(f)** Mosaic augmentation.

**Table 1:** Cultivation-unit-level dataset partitioning across the two cultivation cycles.

Cultivation cycle	Cultivar	Red:blue ratio	Total images	Training images (units)	Validation images (units)	Test images (units)
Cycle 1	Jinpin	8:2	571	571 (all units)	–	–
Cycle 1	Yanqing	9:1	233	233 (all units)	–	–
Cycle 2	Yanqing	8:2	501	168 (7)	215 (9)	118 (6)
Cycle 2	Jinpin	9:1	219	94 (3)	89 (2)	36 (1)
Total	–	–	1524	1066	304	154

#### 2.1.3 Data augmentation

The number of original images was limited because plant factories have restricted cultivation space, and tip-burn occurrence is affected by several factors, including cultivar characteristics, lighting conditions, nutrient solution parameters, and growth stage. To improve the generalization ability of the model and reduce the risk of overfitting, data augmentation was applied to the images during the training stage. The adopted augmentation methods included image flipping, constrained-angle rotation, scaling and translation, HSV color perturbation, and Mosaic augmentation. These operations simulated image changes caused by different shooting angles, lighting variation, leaf position changes, and background interference. As a result, the model could better adapt to complex plant factory conditions. Examples of data augmentation are shown in **Figure 4**.

### 2.2 Improvement of the YOLOv8n model

YOLOv8 is a widely used one-stage object detection model that adopts an anchor-free decoupled detection head and offers advantages in detection accuracy, inference speed, and deployment flexibility. To select a suitable baseline model, YOLOv8n, YOLOv8s, and YOLOv8m were compared in this study, and the results are shown in **Table 2**. YOLOv8m achieved the highest detection accuracy, with mAP@0.5 and mAP@0.5–0.95 of 90.7% and 50.8%, respectively. However, this improvement came with a much higher computational cost. Its parameters, model size, and FLOPs reached 25.8 M, 52.1 MB, and 78.7 G, respectively. In comparison, YOLOv8n achieved an mAP@0.5 of 89.8%, which was only 0.9 percentage points lower than YOLOv8m. At the same time, it contained only 3.0 M parameters, with a model size of 6.3 MB and 8.1 G FLOPs. Considering both detection accuracy and lightweight performance, YOLOv8n was selected as the baseline model in this study.

**Table 2:** The performance and complexity of different YOLOv8 models.

Model	mAP@0.5/%	mAP@0.5-0.95/%	FLOPs/G	Params/M	Model size/MB
YOLOv8n	89.8	49.0	8.1	3.0	6.3
YOLOv8s	90.4	49.4	28.4	11.1	22.6
YOLOv8m	90.7	50.8	78.7	25.8	52.1

The original YOLOv8n mainly consists of three parts: backbone, neck, and head. To improve the detection of small, low-contrast, and occluded tip-burn regions, TB-YOLOv8n introduces AFGCAttention after the SPPF module, GLSA between the backbone and neck, and a P2-enhanced BiFPN neck, as shown in **Figure 5**. These modules enhance channel responses, integrate global and local spatial information, and preserve high-resolution spatial details, respectively.

**Figure 5:**
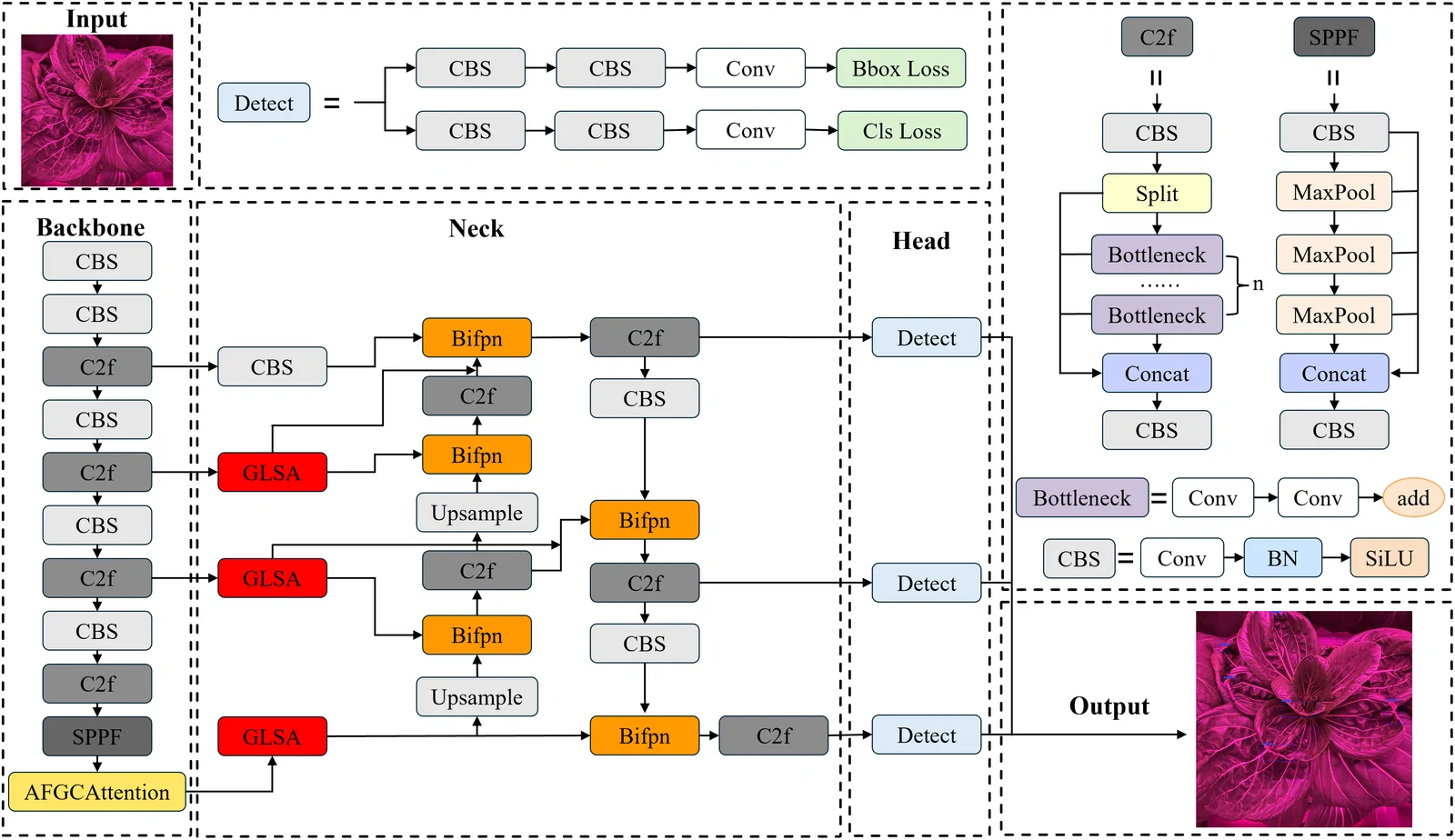
Overall architecture of the proposed TB-YOLOv8n model.

#### 2.2.1 AFGCAttention mechanism

To enhance channel-wise feature representation, Adaptive Fine-Grained Channel Attention (AFGCAttention; Sun et al., 2024), which builds on the SE mechanism (Hu et al., 2018), was introduced at the end of the YOLOv8n backbone. It combines global channel dependency information with local channel interaction information. Through adaptive weight allocation, the module strengthens the feature response of affected regions near leaf tips and margins. It also reduces interference from healthy leaves, leaf vein textures, and light reflections. The structure of the AFGCAttention module is shown in **Figure 6**. The computational process of AFGCAttention is described in Equations 1–8.

**Figure 6:**
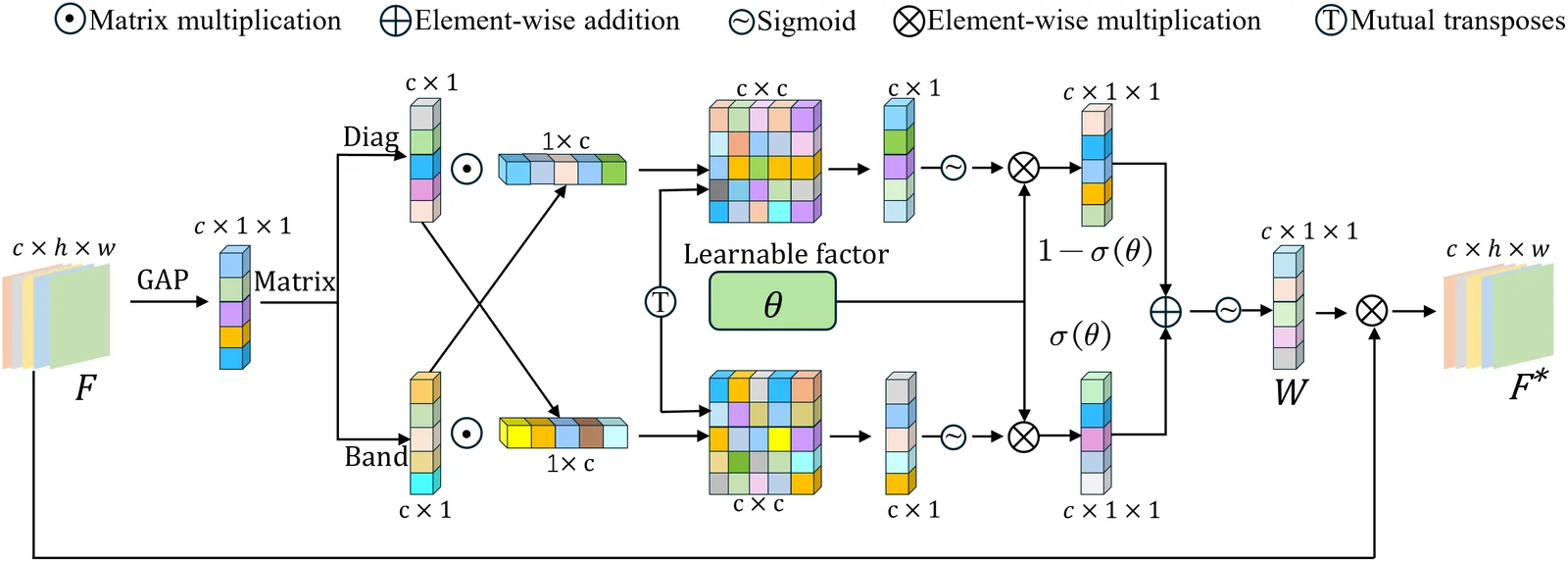
Structure of AFGCAttention module.

Let the input feature map be defined as $\text{F}\in {\text{R}}^{\text{c}\times \text{h}\times \text{w}}$, where c, h, and w denote the number of channels, height, and width of the feature map, respectively. Global average pooling (GAP) is first applied to the input feature map to obtain the channel descriptor (U). The global descriptor of the (n)-th channel can be written as:

(1) $${\text{U}}_{\text{n}}=\text{GAP}\left({\text{F}}_{\text{n}}\right)=\frac{1}{\text{h}\times \text{w}}\sum_{\text{i}=1}^{\text{h}}\sum_{\text{j}=1}^{\text{w}}{\text{F}}_{\text{n}}\left(\text{i},\text{j}\right)$$

where F_n_(i,j) represents the feature value of the n-th channel at spatial position (i,j).

To model local channel interactions while keeping the number of parameters low, AFGCAttention uses a band matrix $\text{B}=\left[{\text{b}}_{1},{\text{b}}_{2},\ldots ,{\text{b}}_{\text{k}}\right]$. This matrix captures dependencies among neighboring channels. The local channel information is calculated as follows:

(2) $${\text{U}}_{\text{lc}}=\sum_{\text{i}=1}^{\text{k}}\text{U}\cdot {\text{b}}_{\text{i}}$$

where U denotes the channel descriptor, U_lc_ denotes the local channel information, k denotes the number of neighboring channels involved in local interaction, and b_i_ represents the i-th column of the band matrix B.

To capture global channel dependencies, a diagonal matrix $\text{D}=\left[{\text{d}}_{1},{\text{d}}_{2},\ldots ,{\text{d}}_{\text{C}}\right]$ is used to obtain global channel information. The calculation is expressed as:

(3) $${\text{U}}_{\text{gc}}=\sum_{\text{i}=1}^{\text{C}}\text{U}\cdot {\text{d}}_{\text{i}}$$

where d_i_ denotes the i-th element of the diagonal matrix D, and U_gc_ represents the global channel information.

After obtaining the local channel information U_lc_ and the global channel information U_gc_, a correlation matrix M is constructed through a cross-correlation operation. This operation enhances the interaction between global and local channel information:

(4) $$\text{M}={\text{U}}_{\text{gc}}\cdot {\text{U}}_{\text{lc}}^{\text{T}}$$

Then, the global channel weights and local channel weights are extracted from the correlation matrix M and its transpose, respectively:

(5) $${\text{U}}_{\text{gc}}^{\text{w}}=\sum_{\text{j}=1}^{\text{c}}{\text{M}}_{\text{i},\text{j}},\text{ i}=1,2,\ldots ,\text{c}$$

(6) $${\text{U}}_{\text{lc}}^{\text{w}}=\sum_{\text{j}=1}^{\text{c}}{\text{M}}_{\text{i},\text{j}}^{\text{T}},\text{ i}=1,2,\ldots ,\text{c}$$

Finally, a learnable factor θ is introduced to adaptively fuse the global and local channel weights, generating the final channel attention weight W:

(7) $$\text{W}=\sigma \left(\sigma \left(\theta \right)\cdot \sigma \left({\text{U}}_{\text{gc}}^{\text{w}}\right)+\left(1-\sigma \left(\theta \right)\right)\cdot \sigma \left({\text{U}}_{\text{lc}}^{\text{w}}\right)\right)$$

where σ(·) denotes the Sigmoid activation function, and θ adaptively controls the contribution ratio between the global channel weights and local channel weights. The final attention weight is multiplied element-wise with the input feature map to obtain the enhanced output feature map:

(8) $${\text{F}}^{*}=\text{W}⊗\text{F}$$

where ⊗ denotes element-wise multiplication, and F^*^ denotes the output feature map enhanced by the AFGCAttention module.

#### 2.2.2 GLSA feature enhancement module

In the multi-scale feature fusion process of YOLOv8n, conventional convolution is usually used to align the channel dimensions of backbone features. This operation ensures that features from different scales can be fused in the neck. However, a single convolution operation has limited ability to describe spatial relationships and local details. This limitation may weaken the fusion between high-level semantic information and low-level detail information. To improve the model’s ability to capture both local details and broader spatial relationships, the Global-to-Local Spatial Aggregation (GLSA) module proposed by Tang et al. (2024) was introduced before the neck.

The structure of the GLSA module is shown in **Figure 7**. The computational process of GLSA is described in Equations 9–14. Let the input feature map be denoted as ${\text{F}}_{\text{in}}\in {\text{R}}^{\text{c}\times \text{h}\times \text{w}}$, where c, h, and w represent the number of channels, height, and width of the input feature map, respectively. First, the GLSA module divides the input feature map into a global-branch input feature ${\text{F}}_{g}^{\text{in}}$ and a local-branch input feature ${\text{F}}_{l}^{\text{in}}$ along the channel dimension. These two feature branches are then fed into the Global Spatial Attention (GSA) branch and the Local Spatial Attention (LSA) branch, respectively:

**Figure 7:**
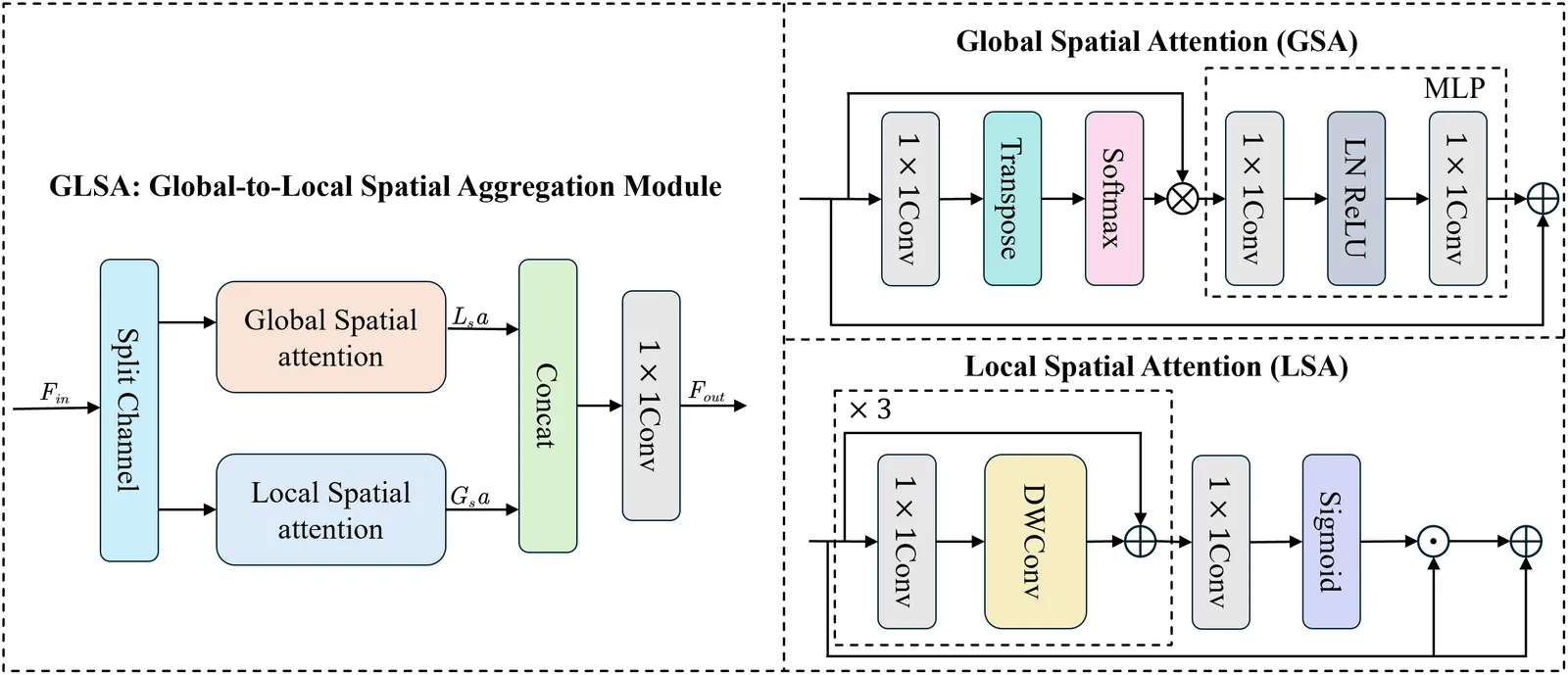
Structure of GLSA module.

(9) $${\text{F}}_{\text{g}}=\text{GSA}\left({\text{F}}_{g}^{\text{in}}\right),{\text{F}}_{\text{l}}=\text{LSA}\left({\text{F}}_{l}^{\text{in}}\right)$$

In the GSA branch, a 1×1 convolution is first applied to transform the input features along the channel dimension. The transformed features are then processed by transpose and Softmax operations to generate global spatial attention weights:

(10) $${\text{A}}_{\text{g}}=\text{Softmax}\left(\text{Transpose}\left({\text{C}}_{1\times 1}\left({\text{F}}_{g}^{\text{in}}\right)\right)\right)$$

where C_1×1_(·) denotes the 1×1 convolution operation, Transpose(·) denotes the feature transpose operation, and Softmax(·) is used for normalization to obtain the global spatial attention weights. The global spatial attention weights are then multiplied with the global-branch input features through matrix multiplication. After that, an MLP is used for feature transformation, and a residual connection is added to preserve the original feature information. The global spatial aggregation feature is calculated as follows:

(11) $${\text{F}}_{\text{g}}=\text{MLP}\left({\text{A}}_{\text{g}}⊗{\text{F}}_{g}^{\text{in}}\right)+{\text{F}}_{g}^{\text{in}}$$

where ⊗ denotes matrix multiplication, MLP(·) denotes a multi-layer perceptron, and the residual connection is used to preserve the original feature information.

In the LSA branch, the input features sequentially pass through a 1×1 convolution and a depthwise convolution (DWConv) to extract local spatial features. A Sigmoid activation function is then used to generate the local spatial attention weights:

(12) $${\text{A}}_{\text{l}}=\text{σ}\left({\text{C}}_{1\times 1}\left(\text{DWConv}\left({\text{C}}_{1\times 1}\left({\text{F}}_{l}^{\text{in}}\right)\right)\right)\right)$$

where σ(·) denotes the Sigmoid activation function. The local spatial attention weights are then multiplied element-wise with the local-branch input features, and a residual connection is introduced to obtain the local spatial aggregation feature:

(13) $${\text{F}}_{\text{l}}={\text{A}}_{\text{l}}⊙{\text{F}}_{l}^{\text{in}}+{\text{F}}_{l}^{\text{in}}$$

where ⊙ denotes element-wise multiplication.

Finally, the output features of the GSA and LSA branches are concatenated along the channel dimension and fused through a 1×1 convolution to obtain the enhanced output feature map:

(14) $${\text{F}}_{\text{out}}={\text{C}}_{1\times 1}\left(\text{Concat}\left({\text{F}}_{\text{g}},{\text{F}}_{\text{l}}\right)\right)$$

where Concat(·) denotes the concatenation operation along the channel dimension, and F_out_ represents the output feature map enhanced by the GLSA module.

#### 2.2.3 Improved BiFPN multi-scale feature fusion

**Figure 8** compares four feature pyramid structures: (a) FPN, (b) PANet, (c) BiFPN, and (d) the improved BiFPN used in this study. In object detection tasks, object scale has a direct effect on feature representation. For pakchoi tip-burn detection, visible affected regions are often small and located near leaf tips or margins. If the model relies only on high-level semantic features, some edge, texture, and location information may be lost. Combining semantic information with spatial details across different scales is therefore important for improving small-region detection.

**Figure 8:**
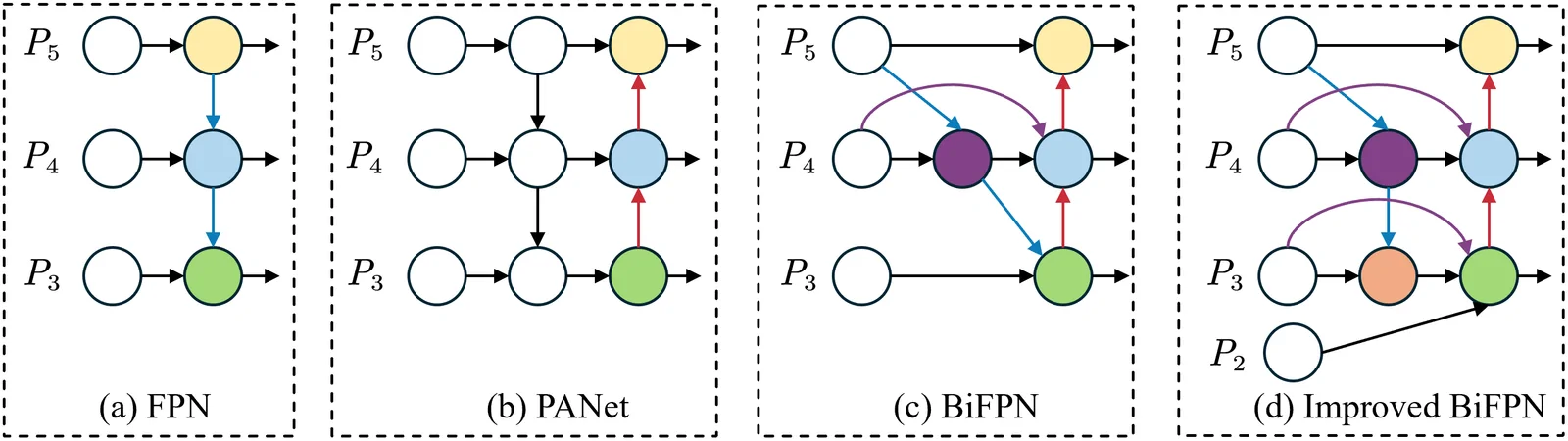
Comparison of different feature pyramid structures: **(a)** FPN; **(b)** PANet; **(c)** BiFPN; **(d)** improved BiFPN.

The traditional Feature Pyramid Network (FPN) (Lin et al., 2017) transfers high-level semantic information to lower-level feature maps through a top-down pathway. PANet (Liu et al., 2018) further adds a bottom-up pathway to strengthen the transfer of low-level location information to higher-level features. However, these structures usually fuse features by simple addition or concatenation, which makes it difficult to adjust the contribution of each scale adaptively. BiFPN (Tan et al., 2020) improves this process by using bidirectional cross-scale connections and learnable weighted fusion. To better preserve spatial details for small tip-burn regions, this study introduces shallow P2 features into BiFPN and constructs an improved BiFPN structure, as shown in **Figure 8d**. Compared with P3, P4, and P5, P2 has higher spatial resolution, retaining more edge, texture, and positional information to provide finer spatial details.

During feature fusion, feature maps from different levels are first aligned in spatial size through upsampling or downsampling. Let the i-th aligned input feature map be ${\text{F}}_{i}^{\prime }$ with a corresponding learnable weight w_i_. The normalized weighted fusion is formulated in Equation (15):

(15) $${\text{F}}_{\text{out}}=\sum_{\text{i}}\frac{{\text{w}}_{\text{i}}}{\in +\sum_{\text{j}}{\text{w}}_{\text{j}}}\cdot {\text{F}}_{i}^{\prime }$$

where w_i_ is the learnable non-negative weight for each input feature map, and *∈* is a small constant to avoid division by zero. In practice, w_i_ is passed through a ReLU activation to ensure non-negativity and normalized to constrain the relative contributions of each input feature. The fused feature map is then refined through a 3×3 convolution to enhance both contextual semantics and local detail representation.

### 2.3 Experimental environment and training strategy

The experimental environment is summarized in **Table 3**. The YOLO-based models were trained for 250 epochs with a batch size of 16 and an input image size of 640 × 640. SGD was used as the optimizer, with an initial learning rate of 0.01, momentum of 0.937, and weight decay of 0.0005. Mosaic augmentation was disabled in the last 10 epochs, and the early stopping patience was set to 50.

**Table 3:** Experimental environment configuration.

Parameters	Configuration
CPU	16-core Intel Xeon Gold 6430
GPU	NVIDIA GeForce RTX 4090
GPU memory size	24 GB
Operating system	Ubuntu 22.04
Deep learning architecture	PyTorch 2.2.2 + Python 3.10 + CUDA 11.8

For the comparative experiments, all models used the same training, validation, and test splits and an input resolution of 640 × 640 pixels. Training configurations were kept consistent whenever applicable, whereas architecture-specific parameters that were not explicitly modified retained the default settings of the corresponding implementations. Official pretrained weights were used for model initialization. The confidence threshold and NMS IoU threshold were set to 0.001 and 0.7, respectively. These settings were used for quantitative evaluation, whereas a confidence threshold of 0.25 was used for the qualitative detection visualizations. To assess the stability of the main performance improvement, YOLOv8n and TB-YOLOv8n were independently trained three times using random seeds of 0, 42, and 100, and their mAP results are reported as mean ± standard deviation.

FPS measurements on both the RTX 4090 and Jetson AGX Orin used 640 × 640 input tensors, a batch size of 1, 200 warm-up iterations, and 1,000 timed forward passes under FP32 precision. CUDA synchronization was applied during timing, and image preprocessing and post-processing were excluded. YOLOv8n and TB-YOLOv8n were additionally tested on an NVIDIA Jetson AGX Orin Developer Kit (64 GB) to evaluate embedded-device inference performance.

### 2.4 Model evaluation metrics

To evaluate the detection performance and model complexity, precision (P), recall (R), mAP@0.5, mAP@0.5–0.95, FPS, the number of model parameters (Params), FLOPs, and model size were used. mAP@0.5–0.95 represents the mean AP averaged over IoU thresholds from 0.50 to 0.95 with a step size of 0.05, providing a stricter evaluation of bounding-box localization accuracy.

The calculation formulas for P, R, AP, mAP@0.5, and mAP@0.5–0.95 are given in Equations 16–20:

(16) $$\text{P}=\frac{\text{TP}}{\text{TP}+\text{FP}}$$

(17) $$\text{R}=\frac{\text{TP}}{\text{TP}+\text{FN}}$$

(18) $$\text{AP}=\int_{0}^{1}\text{P}\left(\text{R}\right)\text{dR}$$

(19) $$\text{mAP}@0.5=\frac{1}{N}\sum_{i=1}^{N}AP_{i}^{0.5}$$

(20) $$\text{mAP}@0.5-0.95=\frac{1}{10N}\sum_{j=0}^{9}\sum_{i=1}^{N}AP_{i}^{0.50+0.05j}$$

where TP represents the number of correctly detected objects, FP represents the number of false detections, and FN represents the number of missed detections. $AP_{i}^{t}$ denotes the average precision of the *i*-th class at an IoU threshold of *t*, and *N* denotes the number of classes. FPS indicates the number of images processed per second, and Params denotes the number of model parameters.

## 3 Results

### 3.1 Comparative experiment of attention mechanisms

To verify the effectiveness of the AFGCAttention module in pakchoi tip-burn detection, YOLOv8n was used as the baseline model. Different attention mechanisms, including CAFM (Hu et al., 2024), DAttention (Xia et al., 2022), MLCA (Wan et al., 2023), MPCA (Mao et al., 2024), and AFGC, were inserted after the SPPF module for comparison under the same experimental settings.

Compared with the baseline model, most attention modules improved precision and recall to varying degrees (**Table 4**). Among all compared methods, YOLOv8n-AFGC achieved the highest mAP@0.5 of 91.7%, which was 1.9 percentage points higher than that of the original YOLOv8n. Its precision and recall reached 87.7% and 86.5%, respectively, representing increases of 2.5 and 2.8 percentage points over the baseline. Different attention mechanisms showed different metric preferences. DAttention achieved the highest recall of 87.1%, while MLCA achieved the highest precision of 88.5%. CAFM increased precision, recall, and mAP@0.5 compared with the baseline model, but its mAP@0.5 was lower than that of AFGC. MPCA increased precision to 87.2%, while its mAP@0.5 reached 90.0%, only 0.2 percentage points higher than the baseline model. In terms of model complexity, YOLOv8n-AFGC introduced almost no additional computational cost. Its FLOPs remained 8.1 G, and the number of parameters remained 3.0 M. The model size increased only slightly, from 6.3 MB to 6.5 MB. In contrast, CAFM, DAttention, and MPCA introduced larger increases in parameters or model size. Overall, YOLOv8n-AFGC achieved the best mAP@0.5 while maintaining nearly the same computational complexity as the baseline model.

**Table 4:** Comparison results of different attention mechanisms.

Model	P/%	R/%	mAP@0.5/%	FLOPs/G	Params/M	Model size/MB
YOLOv8n	85.2	83.7	89.8	8.1	3.0	6.3
YOLOv8n-CAFM	86.5	86.0	90.5	8.4	3.3	7.0
YOLOv8n-DAttention	86.4	87.1	91.6	8.3	3.2	6.9
YOLOv8n-MLCA	88.5	84.3	91.5	8.1	3.0	6.3
YOLOv8n-MPCA	87.2	84.3	90.0	8.1	3.3	7.0
YOLOv8n-AFGC	87.7	86.5	91.7	8.1	3.0	6.5

### 3.2 Comparative experiment of neck network

To evaluate the effect of different neck feature fusion structures on pakchoi tip-burn detection, the original YOLOv8n neck, FPN, BiFPN, and the improved BiFPN-P2 with an additional shallow P2 feature layer were compared under the same experimental settings, with results presented in **Table 5**.

**Table 5:** Comparison results of different neck feature fusion structures.

Model	P/%	R/%	mAP@0.5/%	FLOPs/G	Params/M	Model size/MB
YOLOv8n	85.2	83.7	89.8	8.1	3.0	6.3
YOLOv8n-FPN	90.4	80.4	89.5	6.3	1.8	3.9
YOLOv8n-BiFPN	85.8	86.5	90.4	6.5	1.9	4.1
YOLOv8n-BiFPN-P2	85.9	87.4	90.9	7.1	1.9	4.3

YOLOv8n-FPN greatly reduced model complexity. Its FLOPs, parameters, and model size decreased to 6.3 G, 1.8 M, and 3.9 MB, respectively, while its precision increased to 90.4%. However, its recall dropped to 80.4%, and its mAP@0.5 was slightly lower than that of the baseline model. Compared with FPN, YOLOv8n-BiFPN achieved a better balance between precision and recall, with recall and mAP@0.5 increased to 86.5% and 90.4%, respectively. After introducing the shallow P2 feature layer, YOLOv8n-BiFPN-P2 achieved the best overall balance among the compared neck structures. Its recall and mAP@0.5 reached 87.4% and 90.9%, respectively, which were 3.7 and 1.1 percentage points higher than those of the original YOLOv8n. At the same time, its FLOPs, parameters, and model size remained lower than those of the baseline model.

### 3.3 Ablation experiment

To evaluate the contribution of each improved module to pakchoi tip-burn detection, the original YOLOv8n was used as the baseline model. Under the same experimental settings, the AFGC, GLSA, and BiFPN-P2 modules were introduced individually and in different combinations.

#### 3.3.1 Ablation results and metric visualization

**Table 6** presents the ablation experiment results for different module combinations. Compared with the original YOLOv8n, each module improved detection performance when introduced separately. After adding AFGC, the precision, recall, and mAP@0.5 increased to 87.7%, 86.5%, and 91.7%, respectively. The corresponding mAP@0.5–0.95 increased from 49.0% for the baseline to 53.8%. After introducing GLSA, precision and mAP@0.5 reached 88.1% and 91.4%, respectively, with mAP@0.5–0.95 further increasing to 56.4%. The improved BiFPN-P2 achieved the highest recall among the single-module variants, reaching 87.4%, and its mAP@0.5–0.95 reached 56.2%. It also reduced the parameters and model size to 1.9 M and 4.3 MB, respectively, with FLOPs of 7.1 G.

**Table 6:** Result of ablation experiments.

Baseline	AFGC	GLSA	BiFPN-P2	P/%	R/%	mAP@0.5/%	mAP@0.5-0.95/%	FLOPs/G	FPS	Params/M	Model size/MB
√	×	×	×	85.2	83.7	89.8 ± 0.3	49.0 ± 0.3	8.1	282	3.0	6.3
√	√	×	×	87.7	86.5	91.7	53.8	8.1	234	3.0	6.5
√	×	√	×	88.1	86.7	91.4	56.4	9.2	171	4.0	8.5
√	×	×	√	85.9	87.4	90.9	56.2	7.1	227	1.9	4.3
√	√	√	×	87.2	85.7	92.0	57.5	9.2	156	4.0	8.6
√	√	×	√	83.7	84.6	90.5	58.5	7.1	198	2.0	4.5
√	×	√	√	89.1	86.1	91.3	59.7	7.6	148	2.1	4.7
√	√	√	√	89.4	87.6	93.2 ± 0.3	59.0 ± 0.3	7.6	138	2.2	4.8

The dual-module results further demonstrated the interaction among different improvements. The combination of AFGC and GLSA increased mAP@0.5 to 92.0%, with an mAP@0.5–0.95 of 57.5%, although its precision and recall were slightly lower than those of some single-module settings. The combination of AFGC and improved BiFPN-P2 maintained a lightweight structure, but its improvement in mAP@0.5 was limited, whereas its mAP@0.5–0.95 increased to 58.5%. In contrast, GLSA combined with improved BiFPN-P2 increased precision to 89.1% while keeping the model compact. This combination achieved the highest mAP@0.5–0.95 among the ablation configurations, reaching 59.7%. When all three modules were introduced simultaneously, the complete TB-YOLOv8n model achieves the best overall balance, with the precision, recall, and mAP@0.5 reaching 89.4%, 87.6%, and 93.2%, respectively. Its mAP@0.5–0.95 reached 59.0%, representing a 10.0 percentage point improvement over the 49.0% of the baseline YOLOv8n. Although the GLSA + BiFPN-P2 combination achieved a slightly higher mAP@0.5–0.95 (59.7%), the complete model achieved a better overall balance among precision, recall, mAP@0.5, strict localization performance, and model complexity. The model contained 2.2 M parameters, 7.6 G FLOPs, a size of 4.8 MB, and an inference speed of 138 FPS, balancing detection accuracy, lightweight design, and real-time capability.

**Figure 9** further compares the Precision–Recall and F1–confidence curves of YOLOv8n and TB-YOLOv8n. The Precision–Recall curve of TB-YOLOv8n was generally closer to the upper-right corner, indicating a more favorable precision–recall trade-off. In addition, the maximum F1 score increased from 0.85 for YOLOv8n to 0.88 for TB-YOLOv8n, while the corresponding optimal confidence thresholds remained similar (0.399 for YOLOv8n and 0.379 for TB-YOLOv8n). These results further demonstrate that the proposed model achieves a better balance between precision and recall.

**Figure 9:**
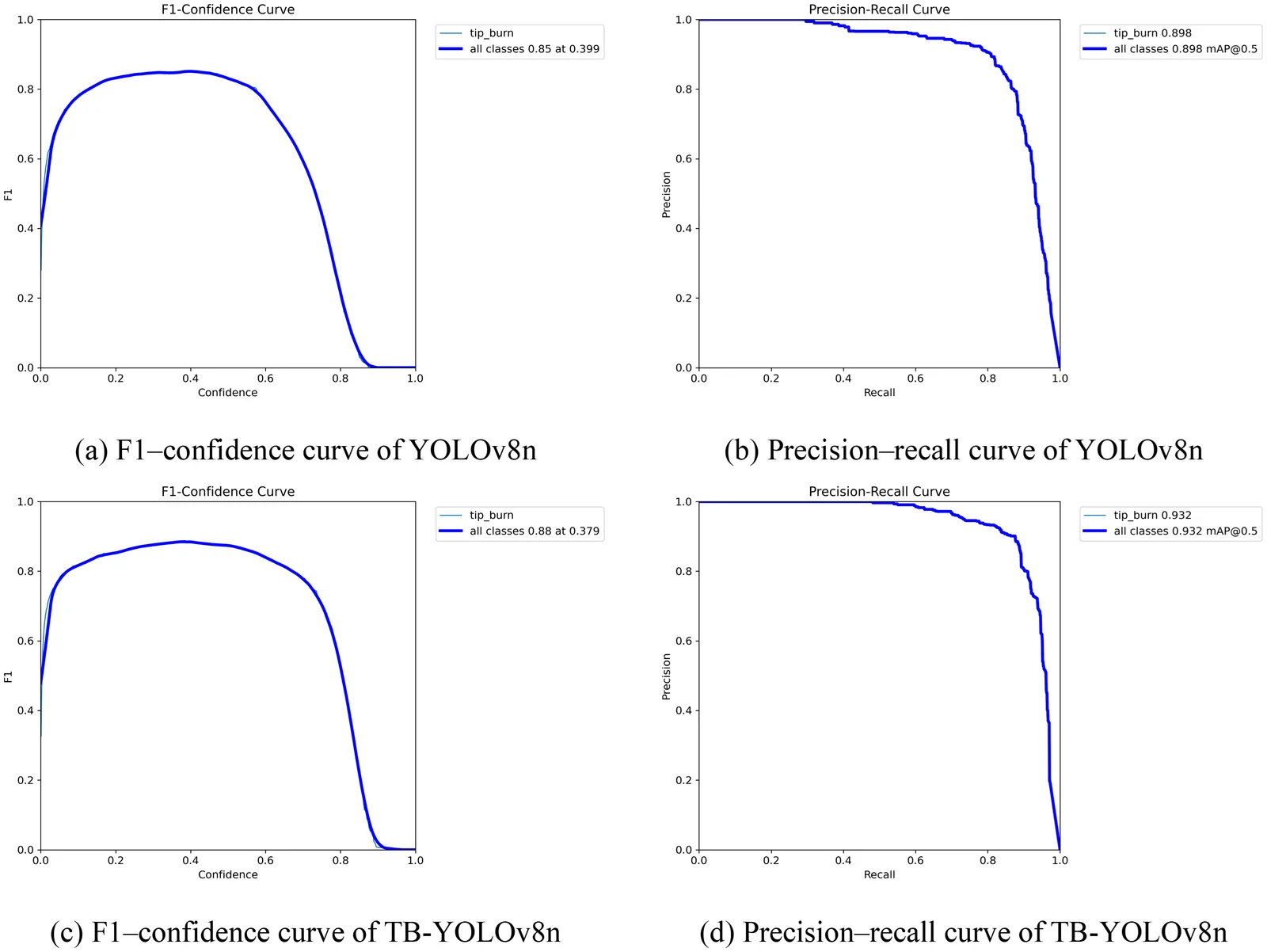
Precision–Recall and F1–confidence curves of YOLOv8n and TB-YOLOv8n. **(a)** F1–confidence curve of YOLOv8n; **(b)** Precision–Recall curve of YOLOv8n; **(c)** F1–confidence curve of TB-YOLOv8n; **(d)** Precision–Recall curve of TB-YOLOv8n.

#### 3.3.2 Visualization analysis of heatmaps

To further analyze the feature response of the improved model to tip-burn regions, Grad-CAM++ (Chattopadhay et al., 2018) was used to visualize the activation regions of YOLOv8n and TB-YOLOv8n (**Figure 10**).

**Figure 10:**
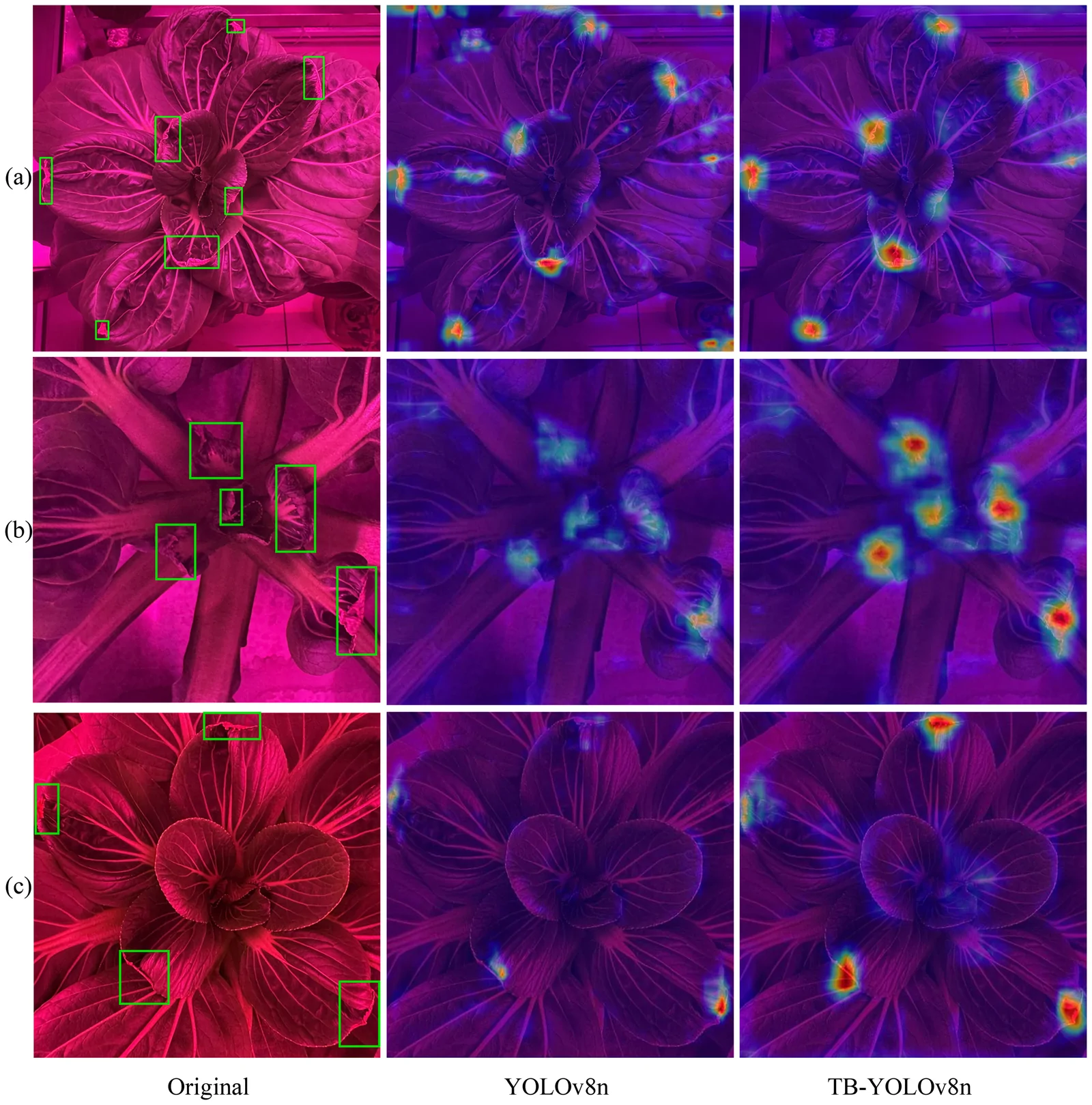
Heatmap comparison between YOLOv8n and TB-YOLOv8n. **(a)** Leaf overlap and margin curling; **(b)** heart-leaf occlusion; **(c)** small-area marginal tip-burn.

For samples with obvious leaf overlap and curled leaf margins, the activation regions of YOLOv8n were relatively scattered, and some responses appeared on healthy leaves or background areas. In contrast, TB-YOLOv8n produced more concentrated activation responses around tip-burn regions near the leaf margins (**Figure 10a**). For samples with tip-burn near the heart leaves (**Figure 10b**), the affected regions were easily confused with young leaves, petiole shadows, and compact heart-leaf structures. YOLOv8n showed less focused responses in these areas, while TB-YOLOv8n generated stronger activation around the heart leaves and nearby tip-burn regions. For samples collected under a higher proportion of red light, the affected regions were relatively small and had weak visual contrast (**Figure 10c**). YOLOv8n showed weak responses to subtle marginal tip-burn, whereas TB-YOLOv8n formed clear activation regions along the affected leaf edges.

#### 3.3.3 Visualization analysis of detection results

To visually compare the detection performance of the original YOLOv8n and TB-YOLOv8n in complex scenarios, representative samples were selected under different red-blue light ratios, leaf morphologies, and occlusion conditions (**Figure 11**). These samples included the Yanqing–8:2 and Jinpin–9:1 cultivar–lighting combinations represented in the test set. All predictions were visualized using a consistent confidence threshold.

**Figure 11:**
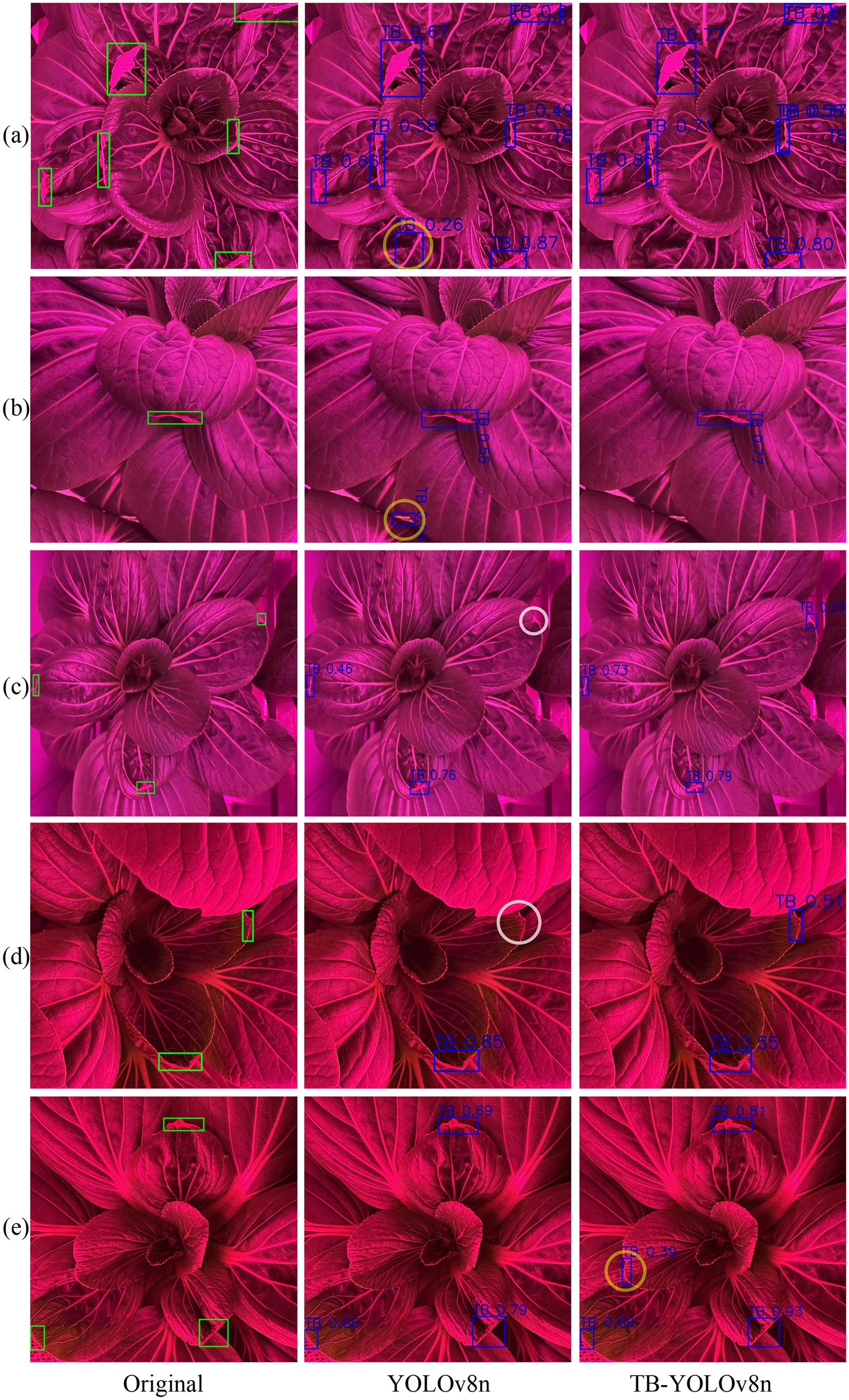
Detection result comparison between YOLOv8n and TB-YOLOv8n. **(a–c)** Yanqing samples collected under the red–blue light ratio of 8:2; **(d–e)** Jinpin samples collected under the red–blue light ratio of 9:1. Yellow circles indicate false detections, and white circles indicate missed detections.

Under the red-blue light ratio of 8:2, some tip-burn regions were light in color and showed only weak differences from normal leaf margins, vein textures, or background areas (**Figures 11a–c**). YOLOv8n misidentified some healthy leaf textures or background regions as tip-burn (**Figures 11a, b**). For small visible tip-burn regions located at leaf margins, the baseline model also produced missed detections (**Figure 11c**). In contrast, TB-YOLOv8n more accurately distinguished affected regions from normal leaf textures and effectively localized small marginal tip-burn regions (**Figures 11a–c**). Under the red-blue light ratio of 9:1, the images were more strongly affected by red light (**Figures 11d, e**). When affected regions were partly occluded by leaves and showed low contrast with the background, the baseline model also missed some targets (**Figure 11d**). TB-YOLOv8n correctly localized these low-contrast and partially occluded regions, but it was not free from errors. As shown in **Figure 11e**, a locally highlighted healthy leaf region under red LED illumination was incorrectly identified as tip-burn. This failure indicates that strong local illumination may still produce visual features similar to visible tip-burn symptoms.

### 3.4 Comparison with different models

To further evaluate the effectiveness of the proposed model, TB-YOLOv8n was compared with mainstream object detection models, including Faster R-CNN, YOLOv5n, YOLOv8n, YOLOv10n, and YOLOv12n.

#### 3.4.1 Comparative analysis of detection models

The performances of different object detection models for pakchoi tip-burn detection were summarized in **Table 7**. Faster R-CNN achieved the highest recall of 91.8%, but its precision was only 71.9%. It also had the highest model complexity, with 41.35 M parameters, 90.9 G FLOPs, and a model size of 165 MB. Its inference speed was 69 FPS, which was lower than that of most lightweight YOLO models.

**Table 7:** Comparison results with different detection methods.

Model	P/%	R/%	mAP@0.5/%	mAP@0.5-0.95/%	FLOPs/G	FPS	Params/M	Model size/MB
Faster R-CNN	71.9	91.8	89.3	47.2	90.9	69	41.35	165
YOLOv5n	86.5	83.5	88.5	55.9	7.1	189	2.5	5.3
YOLOv8n	85.2	83.7	89.8 ± 0.3	49.0 ± 0.3	8.1	282	3.0	6.3
YOLOv10n	82.9	83.5	88.0	54.8	6.5	126	2.2	5.8
YOLOv12n	87.3	84.6	90.8	56.4	6.3	90	2.5	5.2
TB-YOLOv8n	89.4	87.6	93.2 ± 0.3	59.0 ± 0.3	7.6	138	2.2	4.8

Among the lightweight YOLO models, YOLOv8n achieved the fastest inference speed, reaching 282 FPS. Its precision, recall, and mAP@0.5 were 85.2%, 83.7%, and 89.8%, respectively. Its mAP@0.5–0.95 was 49.0%. YOLOv5n achieved an mAP@0.5 of 88.5%, with 2.5 M parameters and an inference speed of 189 FPS. YOLOv10n had 2.2 M parameters, but its precision and mAP@0.5 were 82.9% and 88.0%, respectively. YOLOv12n achieved relatively high precision and mAP@0.5, reaching 87.3% and 90.8%, while its recall was 84.6% and its inference speed was 90 FPS. Its mAP@0.5–0.95 reached 56.4%. In comparison, TB-YOLOv8n achieved the best overall detection accuracy among all compared models. Its precision, recall, and mAP@0.5 reached 89.4%, 87.6%, and 93.2%, respectively. Its mAP@0.5–0.95 reached 59.0%, which was the highest among all compared models. Compared with YOLOv8n, these values increased by 4.2, 3.9, and 3.4 percentage points, respectively. Moreover, mAP@0.5–0.95 increased from 49.0% to 59.0%, representing an improvement of 10.0 percentage points under the stricter IoU-based evaluation. Compared with YOLOv12n, TB-YOLOv8n improved mAP@0.5 by 2.4%. Its mAP@0.5 was also 4.7% and 5.2% higher than those of YOLOv5n and YOLOv10n, respectively.

In terms of model complexity, TB-YOLOv8n contained only 2.2 M parameters, which was lower than YOLOv5n, YOLOv8n, and YOLOv12n, and comparable to YOLOv10n. Its FLOPs were 7.6 G, lower than those of YOLOv8n and much lower than those of Faster R-CNN. The model size was 4.8 MB, which was the smallest among all compared models. Although its inference speed of 138 FPS was lower than that of YOLOv8n, YOLOv5n, and YOLOv10n, it was higher than those of Faster R-CNN and YOLOv12n. For the radar chart, precision, recall, mAP@0.5, and FPS were normalized using conventional min–max normalization, whereas the number of parameters and model size were reverse-normalized. Therefore, a larger normalized value represents better performance for all dimensions. As shown in **Figure 12**, TB-YOLOv8n showed a relatively balanced performance in detection accuracy, model compactness, and inference speed. On the Jetson AGX Orin platform, YOLOv8n achieved 61.4 FPS, whereas TB-YOLOv8n achieved 33.0 FPS under the same inference settings. Although the additional feature-enhancement and multi-scale fusion modules reduced inference speed relative to the baseline, TB-YOLOv8n maintained a model-forward inference rate above 30 FPS on the embedded platform. This result provides preliminary experimental evidence for its computational feasibility on edge hardware.

**Figure 12:**
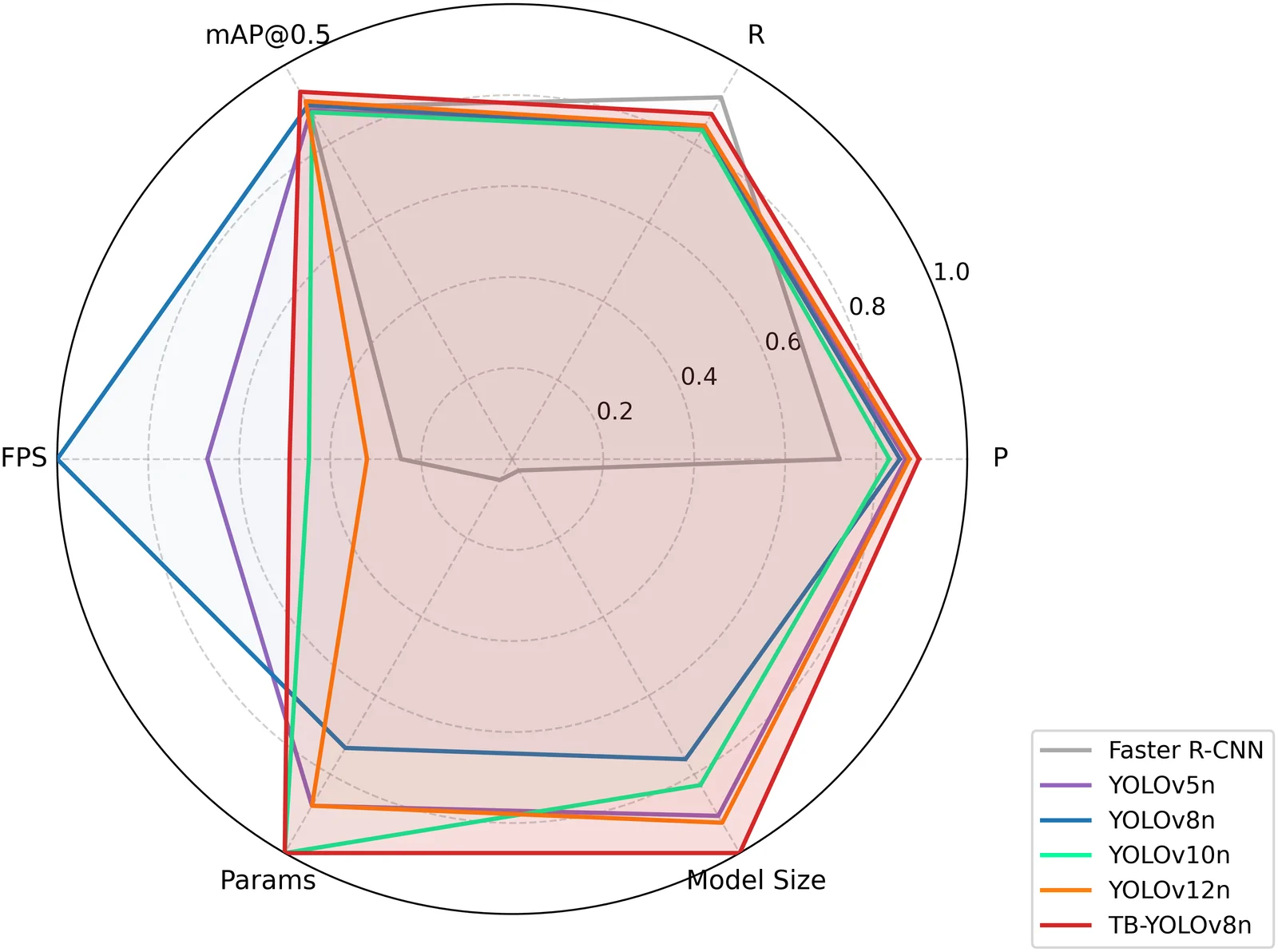
Radar chart comparison of different detection models.

#### 3.4.2 Comparison of detection results

**Figure 13** compares the detection results of different models. The first column shows the ground-truth annotations, while the second to fifth columns show the results of YOLOv5n, YOLOv10n, YOLOv12n, and TB-YOLOv8n, respectively. White circles indicate missed detections.

**Figure 13:**
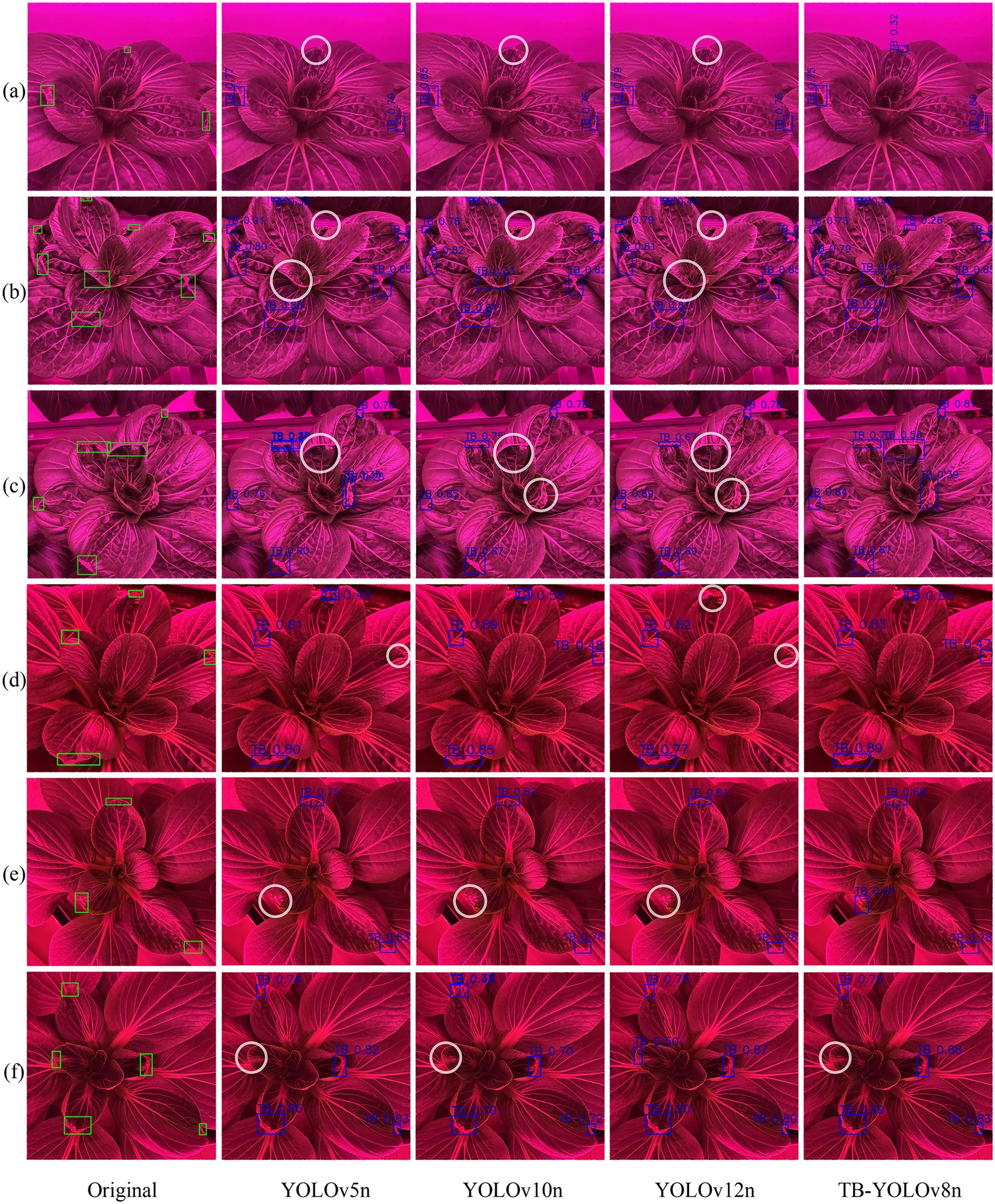
Detection result comparison of different models for pakchoi tip-burn detection. **(a)** Small low-contrast marginal tip-burn; **(b)** tip-burn under leaf overlap; **(c)** elongated marginal tip-burn with curled leaf edges; **(d, e)** small or partially hidden tip-burn under different lighting and leaf morphology conditions; **(f)** a difficult case under strong red-light interference.

To evaluate model performance under real plant factory conditions, representative samples with different leaf shapes, visible symptom appearances, and occlusion levels were selected for visual comparison. The models showed clear differences under red-blue lighting, leaf overlap, and small tip-burn regions (**Figure 13**). The affected region was located near the leaf edge, with a small area and weak color contrast (**Figure 13a**). YOLOv5n, YOLOv10n, and YOLOv12n failed to detect this region. Overlapping leaves caused the affected region to blend with leaf veins and shadowed areas, which led to missed detections by some comparison models (**Figure 13b**). **Figure 13c** shows a sample with curled leaf edges and elongated tip-burn regions near the leaf margins. In this case, the comparison models showed less stable localization of the affected regions. **Figures 13d, e** show samples under different lighting and leaf morphology conditions. Some affected regions were small, hidden near leaf edges or inner leaves, and showed low contrast with the background. YOLOv5n, YOLOv10n, and YOLOv12n had difficulty detecting these hidden tip-burn regions. In general, TB-YOLOv8n provided more stable detection results in these complex scenarios. However, as shown in **Figure 13f**, it still produced a missed detection under strong red-light interference, where the small affected region was visually mixed with nearby leaf veins and background structures.

## 4 Discussion

### 4.1 Effectiveness analysis of model improvements

The improvements to TB-YOLOv8n were designed to address the main visual challenges of tip-burn detection in plant factories, including small affected regions, weak color contrast, leaf overlap, heart-leaf occlusion, and red-blue lighting interference. A comparison of attention mechanisms revealed divergent performance tendencies among the tested modules. DAttention (Xia et al., 2022) achieved the highest recall, while MLCA (Wan et al., 2023) achieved the highest precision. AFGC achieved the highest mAP@0.5 while introducing almost no additional computational cost. AFGCAttention combines global channel dependencies with local channel interactions, enabling adaptive channel weighting to enhance subtle tip-burn features while maintaining a lightweight structure. This mechanism aligns with the core function of channel attention: strengthening informative feature channels and suppressing redundant responses (Sun et al., 2024). Zhang et al. (2025) integrated AFGCAttention into an enhanced YOLO model for X-ray contraband detection to address complex backgrounds and stacked-item interference. Although their task differs from plant tip-burn detection, both require models to extract weak target features from complex backgrounds. This property is particularly valuable for tip-burn detection, where affected regions are often mixed with healthy leaf margins, vein textures, and light reflections.

Multi-scale feature fusion was another important factor affecting detection performance. Although FPN reduced computational cost and improved precision, its recall decreased compared with the baseline model. This result may be related to the limitation of a single top-down fusion path, which may not sufficiently preserve the low-level localization details required for small-region detection. In contrast, BiFPN improved recall and mAP@0.5, indicating that bidirectional feature interaction was more suitable for this task. Previous studies have shown that feature pyramid structures improve detection by combining high-level semantic information with low-level spatial details, while BiFPN further introduced weighted bidirectional fusion for efficient cross-scale interaction (Lin et al., 2017; Liu et al., 2018; Tan et al., 2020). Similarly, Zhao et al. (2026) incorporated BiFPN into a dual-backbone YOLOv8 segmentation framework for greenhouse tomato ripeness detection to improve multi-scale feature fusion under occlusion and variable lighting. This is consistent with the need to detect small or weakly visible tip-burn regions under leaf overlap and red-blue lighting conditions. In the present study, the improved BiFPN-P2 further introduced shallow high-resolution P2 features into the fusion process, helping retain edge, texture, and position information for small tip-burn regions.

The GLSA module further improved the spatial representation of tip-burn features. Unlike a single convolution operation used for channel alignment, GLSA combines global spatial information with local detail features (Tang et al., 2024). This design is suitable for pakchoi tip-burn detection because visible tip-burn symptoms often appear as small marginal discoloration or necrosis rather than large lesion areas. Under leaf overlap and heart-leaf occlusion, local texture details alone may be insufficient, while broader spatial context can help distinguish affected regions from normal leaf structures. Zhao et al. (2025) used a global and local feature fusion module for small targets in low-contrast underwater detection, further supporting the value of global-local spatial information for weak-feature and small-object detection. The ablation results were not strictly additive. Although the AFGC + BiFPN-P2 combination achieved a lower mAP@0.5 than AFGC alone (90.5% vs. 91.7%), its mAP@0.5–0.95 increased from 53.8% to 58.5%. This metric-dependent pattern may indicate partial feature redundancy or optimization competition between channel enhancement and high-resolution shallow-feature fusion. The addition of GLSA may improve feature complementarity by integrating global and local spatial information; however, this interpretation remains tentative because these interactions were not directly measured.

Overall, the three modules improved the model from complementary perspectives. AFGCAttention enhanced informative channel responses, GLSA strengthened global-local spatial representation, and improved BiFPN-P2 reinforced shallow multi-scale feature fusion. Their combined use enabled TB-YOLOv8n to achieve the best overall balance among precision, recall, mAP@0.5, localization accuracy, and model complexity. Grad-CAM++ and detection visualizations further showed more concentrated activation around tip-burn regions and generally fewer missed and false detections under red-blue lighting, leaf overlap, and low-contrast conditions, consistent with the quantitative results.

### 4.2 Comparison with previous studies and practical implications

TB-YOLOv8n can be considered in the context of previous studies on tip-burn detection in leafy vegetables grown under controlled environments. Hamidon and Ahamed (2022) compared CenterNet, YOLOv4, and YOLOv5 for lettuce tip-burn detection under different LED lighting conditions, and YOLOv5 achieved the best mAP of 82.8%. Their study confirmed the feasibility of YOLO-based tip-burn detection under artificial lighting. However, the work mainly evaluated existing models and did not introduce a task-specific structure for small visible symptom detection. False detections and missed detections were still observed when tip-burn spots were small or overlapped with leaf background regions. Franchetti and Pirri (2022) combined classification and self-supervised segmentation for tip-burn detection in high-density lettuce canopies grown in plant factories. Their method could localize individual plants and further segment small tip-burn regions, providing an important reference for phenotypic analysis and spatial localization. However, this multi-stage workflow is relatively complex and focuses more on fine segmentation than lightweight real-time detection. TB-YOLOv8n focuses on lightweight detection of small, low-contrast, and partially occluded tip-burn regions by introducing AFGCAttention, GLSA, and improved BiFPN-P2. Unlike the multi-stage classification and segmentation framework, it adopts an end-to-end object detection architecture that directly localizes visible tip-burn regions, providing a relatively simple framework for subsequent monitoring and embedded deployment.

Recent studies have further explored the detection of small visible symptoms and lightweight deployment in controlled-environment crop production. Lin et al. (2026) proposed RT-DETR-TB for leafy vegetable tip-burn detection, improving multi-scale feature fusion and edge feature representation through StarNet, SAFF, and CSEE. Their method mainly focused on strengthening the representation of small tip-burn targets through multi-scale and edge-feature enhancement. Chutichaimaytar et al. (2025) developed CTB-YOLO for visible symptom detection of coriander tip-burn and powdery mildew under indoor conditions and integrated it with a Raspberry Pi edge device for grower notification. This work further demonstrated the application of lightweight disease detection on an embedded platform. In comparison, TB-YOLOv8n focuses on lightweight lesion-level localization of pakchoi tip-burn under red-blue LED lighting, with particular emphasis on small-region feature enhancement, global-local spatial representation, and shallow multi-scale feature fusion.

Compared with the evaluated detection models, TB-YOLOv8n provided a favorable balance between detection accuracy and model complexity. The results suggest that task-oriented channel enhancement, global-local spatial aggregation, and shallow multi-scale fusion are beneficial for detecting small and low-contrast visible tip-burn regions.

At the current stage, TB-YOLOv8n operates at the lesion level and outputs bounding boxes and confidence scores for visible tip-burn regions rather than directly generating plant-level alarms. In practical applications, lesion detections could be associated with individual plants or cultivation units and combined over repeated observations to support plant-level monitoring. The appropriate false-alarm tolerance, monitoring interval, and alarm criteria still require further validation according to actual production requirements. The detection results could also provide decision-support information for checking airflow, lighting, or nutrient-solution conditions when tip-burn occurrence increases. However, automatic adjustment of ventilation, lighting, or nutrient management based on model detections was not investigated in the present study. Overall, TB-YOLOv8n achieved reliable lesion-level localization of visible pakchoi tip-burn symptoms while maintaining a lightweight structure and efficient inference. The proposed feature-enhancement and multi-scale fusion strategies improved the detection of small, low-contrast, and partially occluded tip-burn regions under red-blue LED lighting, providing a basis for future plant-level monitoring and production decision support.

### 4.3 Limitations and future work

Although TB-YOLOv8n achieved good detection performance for pakchoi tip-burn in plant factories, several limitations remain. The current dataset covered a limited range of cultivars, lighting conditions, cultivation cycles, and imaging devices. Although images from the same plant did not cross the training, validation, and test subsets, different cultivation units from the same cultivation cycle were included in these subsets. Therefore, potential correlations associated with shared cultivation conditions cannot be completely excluded, and the current test set should not be regarded as a fully independent cultivation-batch external validation set. In addition, only two negative images were included in the test set, limiting the evaluation of false-positive behavior on fully healthy samples. Imaging angle, occlusion degree, and symptom severity were not recorded as predefined categorical metadata, preventing systematic quantitative subgroup evaluation. Because image acquisition began after visible tip-burn symptoms appeared, the present study evaluates visible symptom detection rather than pre-symptomatic prediction or physiological early warning.

Future work will evaluate the model using a fully independent cultivation cycle or batch and expand the dataset to include more cultivars, lighting conditions, production environments, and negative samples. Structured metadata for imaging angle, occlusion level, and symptom severity will also be recorded to support stratified evaluation. Multispectral or hyperspectral imaging and environmental sensor data may be combined with visible-light images to explore pre-symptomatic physiological stress detection. Further optimization through pruning, quantization, and TensorRT acceleration will also be investigated to improve embedded-device deployment performance.

## 5 Conclusions

To achieve rapid, accurate, and lightweight detection of pakchoi tip-burn in plant factories, this study proposed an improved YOLOv8n-based model named TB-YOLOv8n. The model integrated AFGCAttention, GLSA, and an improved BiFPN-P2 structure with shallow P2 features. These improvements enhanced the representation of small and low-contrast tip-burn regions near leaf tips, leaf margins, and heart leaves.

The experimental results showed that TB-YOLOv8n achieved favorable overall performance in pakchoi tip-burn detection. Its precision, recall, and mAP@0.5 reached 89.4%, 87.6%, and 93.2%, respectively, which were 4.2, 3.9, and 3.4 percentage points higher than those of the original YOLOv8n. Its mAP@0.5–0.95 reached 59.0%, representing an improvement of 10.0 percentage points over the baseline. At the same time, the model contained only 2.2 M parameters, required 7.6 G FLOPs, had a model size of 4.8 MB, and reached model-forward inference speed of 138 FPS on the RTX 4090. On the Jetson AGX Orin platform, it achieved a model-forward inference speed of 33.0 FPS, providing preliminary evidence of its feasibility for embedded deployment. These results indicate that TB-YOLOv8n achieved a favorable balance among detection accuracy, model complexity, and inference efficiency.

Overall, TB-YOLOv8n effectively localized visible tip-burn regions in pakchoi grown under plant factory conditions. The proposed model provides a basis for visible tip-burn monitoring and subsequent plant-level production decision support. Future work will expand multi-scenario datasets, evaluate the model using independent cultivation batches, incorporate environmental sensor information, and further optimize the model for deployment on edge computing platforms to improve its applicability in real-time plant factory monitoring systems.

## Data Availability

The raw data supporting the conclusions of this article will be made available by the authors, without undue reservation.
