# Supplementary material for: TB-YOLOv8n: an improved YOLOv8n-based model for tip-burn detection in plant factory-grown pakchoi under LED lighting
Source: Front Plant Sci. 2026 Sep 23;17:1933084. doi: 10.3389/fpls.2026.1933084 (PMC13642603; doi:10.3389/fpls.2026.1933084)
Supplement: Supplementary file 1 [file Table1.docx]

Supplementary Material

# 1 Supplementary Figures and Tables

**Table S1.** Dataset composition and annotation statistics.

**Panel A.** Dataset split

| **Dataset split** | **Images** | **Positive images** | **Negative images** | **Bounding-box annotations** |
| --- | --- | --- | --- | --- |
| Training | 1,066 | 1,040 | 26 | 3,213 |
| Validation | 304 | 299 | 5 | 883 |
| Test | 154 | 152 | 2 | 464 |
| **Total** | **1,524** | **1,491** | **33** | **4,560** |

**Panel B.** Cultivar and lighting condition

| **Cultivar** | **Red:blue ratio** | **Images** | **Bounding-box annotations** |
| --- | --- | --- | --- |
| Jinpin | 8:2 | 571 | 1,720 |
| Jinpin | 9:1 | 219 | 642 |
| Yanqing | 8:2 | 501 | 1,503 |
| Yanqing | 9:1 | 233 | 695 |
| **Total** | – | **1,524** | **4,560** |

**Note:** Positive images contain at least one annotated tip-burn region, whereas negative images contain no visible tip-burn target and therefore have no bounding-box annotations.

**Table S2.** Implementation, training, and inference settings of the compared models.

**Panel A.** Model implementation, training, and inference settings

| **Model** | **Implementation source** | **Backbone / architecture** | **Pretrained weights** | **Input size** | **Epochs** | **Augmentation** | **Confidence threshold** | **NMS IoU** |
| --- | --- | --- | --- | --- | --- | --- | --- | --- |
| Faster R-CNN | MMDetection | ResNet-50-FPN | ImageNet-pretrained ResNet-50 (torchvision) | 640 × 640 | 24 | RandomFlip (p=0.5) | 0.05 | 0.5 |
| YOLOv5n | Official implementation | YOLOv5n | Official pretrained weights | 640 × 640 | 250 | Base/default augmentation configuration | Default / base setting | Default / base setting |
| YOLOv8n | Ultralytics | YOLOv8n | Official pretrained weights | 640 × 640 | 250 | Same augmentation strategy as described in Section 2.1.2 | Base setting | Base setting |
| YOLOv10n | Official implementation | YOLOv10n | Official pretrained weights | 640 × 640 | 250 | Base/default augmentation configuration | Default / base setting | Default / base setting |
| YOLOv12n | Official implementation | YOLOv12n | Official pretrained weights | 640 × 640 | 250 | Base/default augmentation configuration | Default / base setting | Default / base setting |
| TB-YOLOv8n | Modified from YOLOv8n | Proposed TB-YOLOv8n | Same initialization as YOLOv8n | 640 × 640 | 250 | As described in Section 2.1.2 | Base setting | Base setting |

**Panel B.** Software environment

| **Model** | **Software / implementation** |
| --- | --- |
| Faster R-CNN | MMDetection **3.3.0** |
| YOLOv5n | Ultralytics **8.4.26** |
| YOLOv8n | Ultralytics **8.2.50** |
| YOLOv10n | Ultralytics **8.4.26** |
| YOLOv12n | Ultralytics **8.4.26** |
| TB-YOLOv8n | Modified from Ultralytics **8.2.50** |
